# Bioassay-based *Corchorus capsularis* L. leaf-derived β-sitosterol exerts antileishmanial effects against *Leishmania donovani* by targeting trypanothione reductase

**DOI:** 10.1038/s41598-020-77066-2

**Published:** 2020-11-24

**Authors:** Pijush Kanti Pramanik, Sajal Chakraborti, Angshuman Bagchi, Tapati Chakraborti

**Affiliations:** Department of Biochemistry and Biophysics, University of Kalyani, Kalyani, 741235 West Bengal India

**Keywords:** Mechanism of action, Natural products, Pharmacology, Biochemistry, Chemical biology, Computational biology and bioinformatics, Drug discovery

## Abstract

Leishmaniasis, a major neglected tropical disease, affects millions of individuals worldwide. Among the various clinical forms, visceral leishmaniasis (VL) is the deadliest. Current antileishmanial drugs exhibit toxicity- and resistance-related issues. Therefore, advanced chemotherapeutic alternatives are in demand, and currently, plant sources are considered preferable choices. Our previous report has shown that the chloroform extract of *Corchorus capsularis* L. leaves exhibits a significant effect against *Leishmania donovani* promastigotes. In the current study, bioassay-guided fractionation results for *Corchorus capsularis* L. leaf-derived β-sitosterol (β-sitosterol_CCL_) were observed by spectroscopic analysis (FTIR, ^1^H NMR, ^13^C NMR and GC–MS). The inhibitory efficacy of this β-sitosterol_CCL_ against *L. donovani* promastigotes was measured (IC_50_ = 17.7 ± 0.43 µg/ml). β-Sitosterol_CCL_ significantly disrupts the redox balance via intracellular ROS production, which triggers various apoptotic events, such as structural alteration, increased storage of lipid bodies, mitochondrial membrane depolarization, externalization of phosphatidylserine and non-protein thiol depletion, in promastigotes. Additionally, the antileishmanial activity of β-sitosterol_CCL_ was validated by enzyme inhibition and an in silico study in which β-sitosterol_CCL_ was found to inhibit *Leishmania donovani* trypanothione reductase (*Ld*TryR). Overall, β-sitosterol_CCL_ appears to be a novel inhibitor of *Ld*TryR and might represent a successful approach for treatment of VL in the future.

## Introduction

Leishmaniasis, the vector-borne neglected tropical disease, is the second leading infectious disease after malaria. Approximately 350 million people are affected by leishmaniasis per year in 98 countries worldwide^1^. Leishmaniasis is caused by parasites of the genus *Leishmania,* which are transmitted to mammals by the bite of the vector, the female *Phlebotominae* sand fly. *Leishmania* parasites exist in two morphological stages: promastigotes, an elongated, flagellated, extracellular form, and amastigotes, an intracellular, round, nonflagellated form^2^. Major clinical manifestations of leishmaniasis comprise cutaneous, mucocutaneous and visceral leishmaniasis (VL). The most dreadful form of the disease is VL, which is caused by the species *Leishmania donovani*^3^.

The current treatment strategy for VL has various limitations and is still under debate due to the lack of suitable vaccines and inadequate chemotherapeutics. The foremost line of recommended therapeutics are pentavalent antimonials, such as sodium stibugluconate (Pentostam) and meglumine antimoniate (Glucantime), which emerged as the only remedies for the disease^4^. Regrettably, due to prolonged treatment strategies, these drugs are associated with high toxicity and increased incidences of drug resistance^4^. Although other drugs, such as pentamidine, miltefosine and amphotericin B, are very well known drugs for the treatment of VL, they exhibit unsatisfactory results due to toxic effects, high cost and, more importantly, drug resistance. Therefore, the search for economical alternative drugs with the weakest side effects seems imperative for VL treatment.

The history of classic plant-derived medicinal agents indicates their potential in modern medicine for their less lethal effects; hence, screening of many plant crude extracts can provide an array of novel biologically active plant products^5^. Thus, studies on natural products are now a priority in modern biological sciences^6^, and accordingly, innovation of novel, natural antileishmanial compounds has been escalated in the last few years^7^. Several plant-derived compounds have been investigated previously to evaluate their effects on various *Leishmania* spp.^8^. Accordingly, although our previous work showed the antiparasitic effects of the chloroform extract of *Corchorus capsularis* L. leaves on the Indian strain of *L. donovani*^9^, exploration of the leaf-derived active component(s) warrants great attention for the development of alternative remedies for VL. With regard to different disease manifestations, phytosterols have attracted attention for their beneficial role in health^10^. Among various phytosterols, β-sitosterol is predominant in plants and has significant dietary importance^11,12^. In recent years, the abundant plant sterol β-sitosterol has also been examined for its effect on various microbial infections, including parasitic diseases^13–16^. Plant-derived β-sitosterol has previously been tested on different species of *Leishmania* parasites, such as *L. amazonensis*^17^ and *L. tropica*^18^. A mixture of phytosterols containing stigmasterol and β-sitosterol has been reported to possess leishmanicidal activity against *L. infantum chagasi*^19^. Although the effect of these plant-derived β-sitosterols has been studied on various *Leishmania* spp., detailed study of plant β-sitosterol as an antiparasitic agent against *L. donovani* has not been performed until now.

The most promising method of effective drug discovery and development is to target the enzymes involved directly or indirectly in the parasite life cycle during the establishment of infection. Trypanothione reductase (TryR) is a key enzyme of *Leishmania* species that maintains redox homeostasis for the survival of the parasite^20^. Hence, considering the role of TryR, it could be assumed to be an appropriate drug target to treat *L. donovani* infection. During the last few years, medicinal use of natural lead components has been of growing interest for targeting different enzymes of parasites^8^. More importantly, plant products have also been shown to be excellent sources of TryR inhibitors^21^. Considering all of these aspects, the current investigation was undertaken to determine the effect of *Corchorus capsularis* L*.* leaf-derived β-sitosterol (β-sitosterol_CCL_) against *L. donovani* by targeting TryR for the design of a selective therapeutic against VL.

## Results

### ***Corchorus capsularis*** L. leaf-derived β-sitosterol (β-sitosterol_CCL_): bioassay-guided isolation and characterization

**Figure 1:**
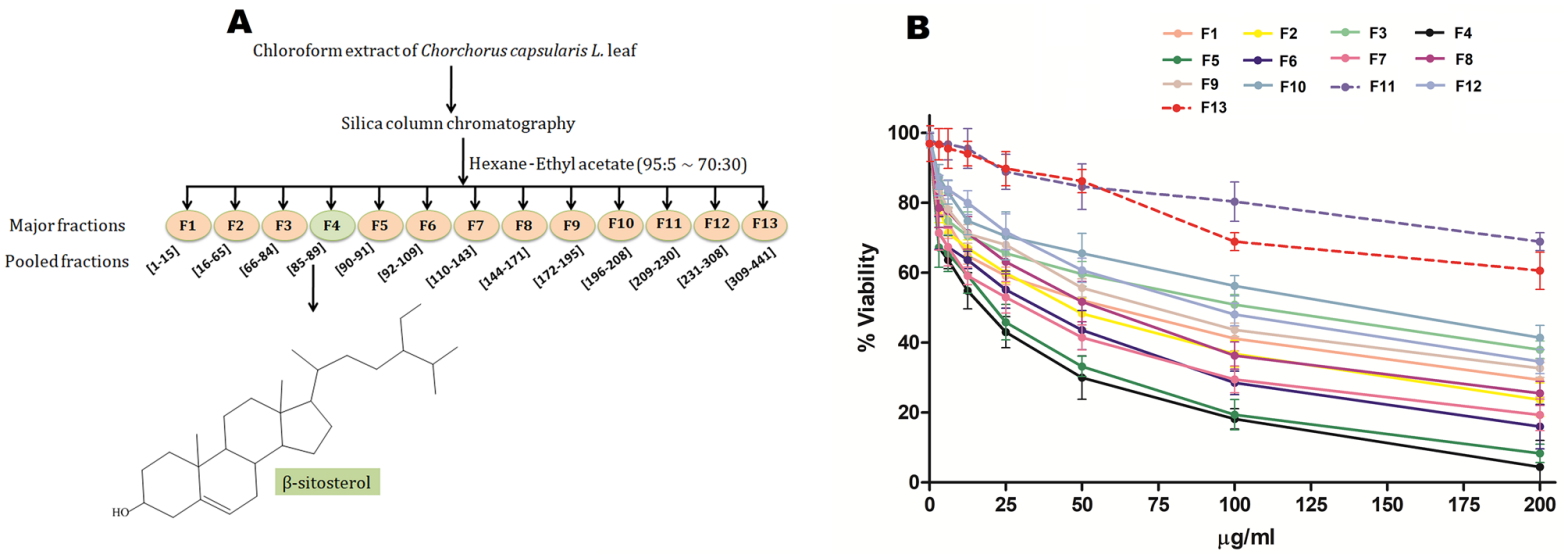
(**A**) Schematic representation of bioassay-guided isolation of β-sitosterol from the chloroform extract of *Corchorus capsularis* L. leaves. (**B**) Determination of the antipromastigote activity of fractions (F1–F13). *L. donovani* promastigotes (1 × 10^7^/ml) were treated individually with 13 fractions (F1–F13) at doses ranging from 0 to 200 µg/ml for 48 h, and the MTT assay was performed. F4 was found to be most active (IC_50_ = 17.7 ± 0.43 µg/ml), denoted by the black line, whereas the F11 and F13 fractions, denoted by dotted lines, exhibited IC_50_ values > 200 µg/ml. The results are expressed as the mean ± S.E. from three independent experiments.

Major fractions (F1 to F13) were obtained according to the schematic presentation shown in Fig. 1A. The antipromastigote activity of all the fractions at doses ranging from 0 to 200 µg/ml was determined by calculating the half-maximal inhibitory concentration (IC_50_) using the MTT assay method (Fig. 1B and Supplementary Table S1). The dose response curve (Fig. 1B) demonstrated that F4 is the most active fraction, having the maximum inhibitory effect with an IC_50_ value of 17.7 ± 0.43 µg/ml. Then, the lead fraction F4 (ethyl acetate:hexane = 7:93) was fully characterized on the basis of spectroscopic analysis (FTIR, ^1^H NMR, ^13^C NMR and GC–MS), the results of which are shown below, and the obtained lead phytocompound (F4) was found to be β-sitosterol.

**Figure 2:**
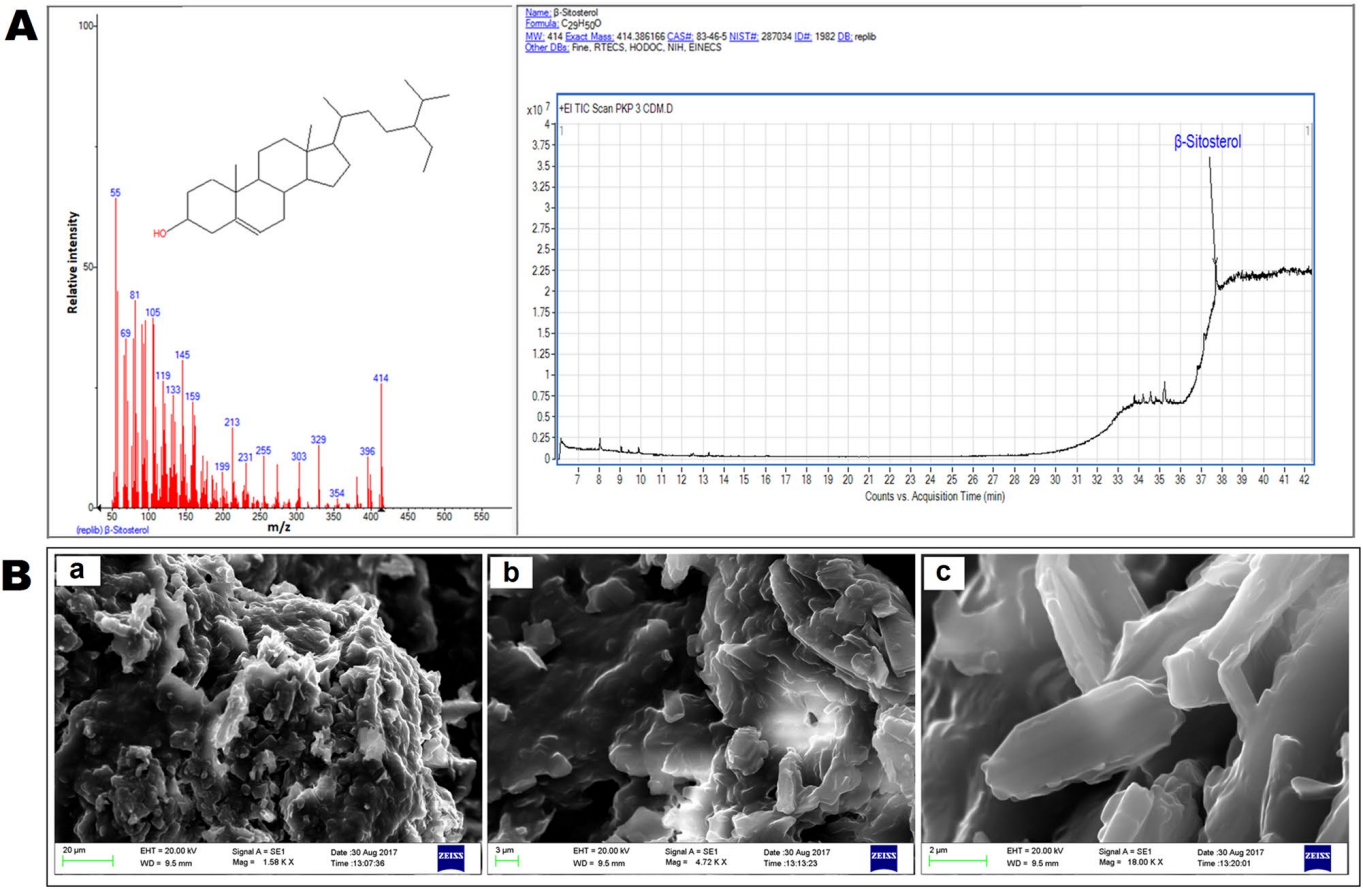
(**A**) Mass spectrum of *Corchorus capsularis* L. leaf-derived β-sitosterol (β-sitosterol_CCL_) is shown in the left panel, and the right panel depicts the chromatogram. GC–MS analysis was performed with a gas chromatograph (Agilent Technologies 7980A) equipped with a mass spectrometric system (7000, GC/MS triple quad). Agilent Mass Hunter software (Version B.50.00) was used for instrument control and data analysis. (**B**) Surface morphology analysis of β-sitosterol_CCL_ by SEM imaging. SEM photographs: (**a**) scale bar, 20 μm; (**b**) scale bar, 3 μm; (**c**) scale bar, 2 μm.

β-Sitosterol (C_29_H_50_O): White yellowish powder, mp 135 °C; FTIR (KBr) ν (cm^−1^): 3423.17, 2955.78, 2918.23, 2849.71, 1737.32, 1706.41, 1463.88, 1166.34 (Supplementary Fig. S1). ^1^H NMR (400 MHz, CDCl_3_): δ 5.340 (s, 1H), 3.559–3.505 (m, 1H), 2.351–2.201 (m, 4H), 2.022–1.949 (m, 3H), 1.840 (d, 3H, *J* = 10 Hz), 1.688–1.587 (m, 3H), 1.561–1.406 (m, 7H), 1.181–1.065 (m, 6H), 1.028–1.005 (m, 5H), 0.963–0.913 (m, 3H), 0.894–0.878 (m, 2H), 0.861–0.843 (m, 4H), 0.823–0.764 (m, 5H), 0.696–0.678 (m, 3H) (Supplementary Fig. S2). ^13^C NMR (100 MHz, CDCl_3_): δ 179.17, 140.69, 121.75, 56.75, 56.04, 50.10, 45.80, 42.15, 39.76, 37.23, 36.49, 36.15, 34.03, 31.89, 31.51, 29.71, 29.68, 29.38, 29.09, 28.25, 26.03, 24.73, 24.30, 22.70, 21.07, 19.39, 19.03, 14.14, 11.85 (Supplementary Fig. S3). The molecular weight obtained by GC–MS was 414 Da (Fig. 2A). In the current study, the *Corchorus capsularis* L. leaf-derived β-sitosterol was designated β-sitosterol_CCL_.

### Surface morphology analysis of β-sitosterol_CCL_

Scanning electron microscopy (SEM) imaging was performed to analyse the surface morphology, and the images obtained by SEM showed the flake-like structure of the isolated leaf compound, as depicted in Fig. 2B.

### Screening of β-sitosterol_CCL_ or commercial β-sitosterol as an antileishmanial agent by antipromastigote activity and cytotoxicity assays

**Figure 3:**
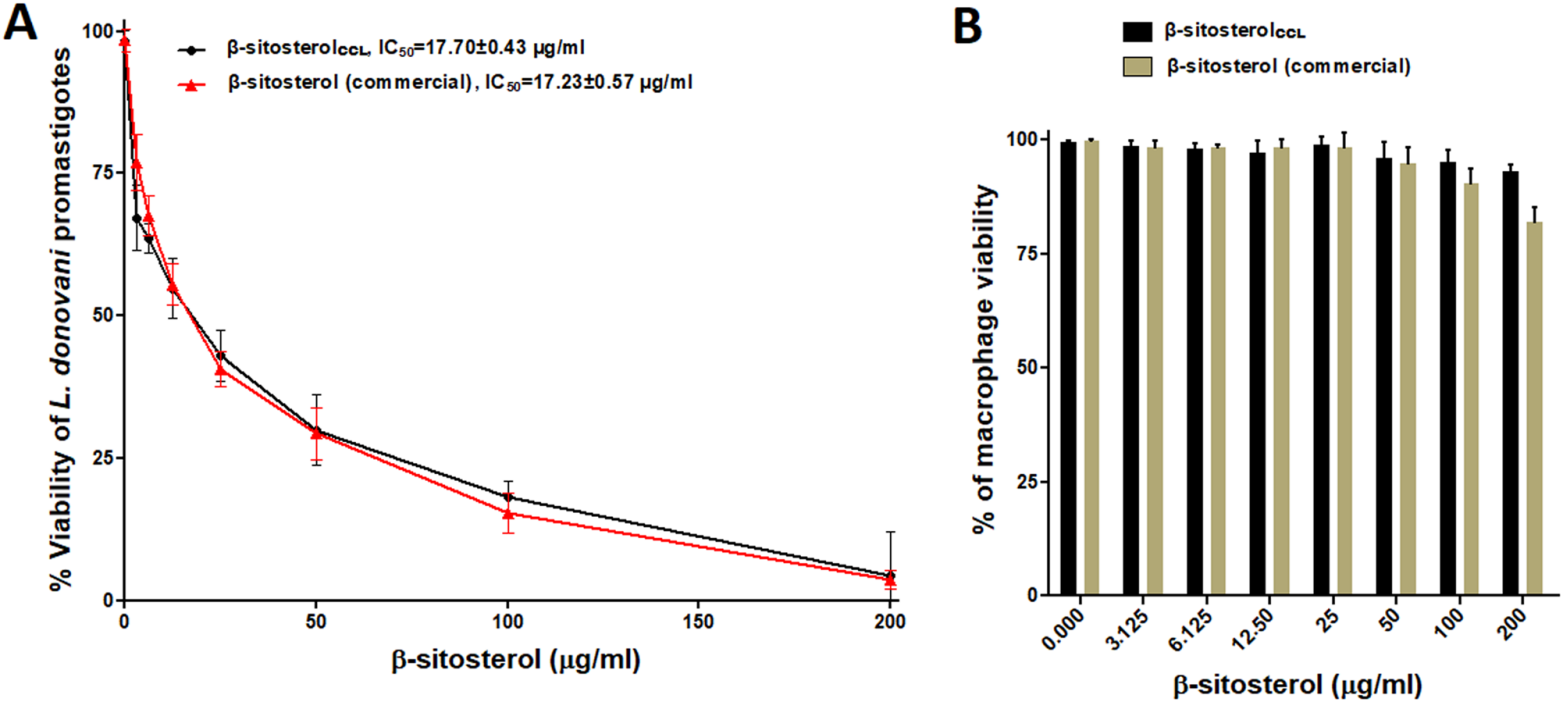
(**A**) Evaluation of antipromastigote activity. *L. donovani* promastigotes (1 × 10^7^/ml) were treated with commercial β-sitosterol (Abcam, USA) (0–200 µg/ml) for 48 h, and the MTT assay was performed, and the IC_50_ was found to be 17.23 ± 0.57 µg/ml, denoted by the red line. β-Sitosterol_CCL,_ as shown by the black line (similar line used in Fig. 1B, IC_50_ = 17.7 ± 0.43 µg/ml), was used for comparison with the commercial standard. The results are presented as the mean ± S.E. from three independent experiments. (**B**) Cytotoxicity against murine RAW 264.7 macrophages. The cytotoxic effects of β-sitosterol_CCL_ and commercial β-sitosterol (Abcam, USA) were tested on macrophages (1 × 10^5^/ml) with increasing concentrations (0–200 µg/ml) for 48 h, and an MTT assay was performed. The results expressed herein are from three independent experiments (mean ± S.E.).

The dose response curve obtained from the MTT assay demonstrated that commercial β-sitosterol (Abcam, USA) exhibits an inhibitory effect against the growth of *L. donovani* promastigotes with an IC_50_ value of 17.23 ± 0.57 µg/ml. Similarly, β-sitosterol_CCL_ showed parasite growth inhibition at IC_50_ = 17.7 ± 0.43 µg/ml (Fig. 3A). These observations clearly demonstrate the analogous efficacy of β-sitosterol_CCL_ compared with that of commercial β-sitosterol in killing *L. donovani* promastigotes.

β-Sitosterol_CCL_ affected 7.04 ± 0.38% of host cells at a high dose (200 µg/ml), but commercial β-sitosterol exhibited greater toxicity at a high dose, exhibiting 18.04 ± 0.75% killing of normal host cells (Fig. 3B). Thus, β-sitosterol_CCL_ displays antileishmanial properties with negligible cytotoxicity even at a high doses. Therefore, β-sitosterol_CCL_ seems to be more reliable than commercial β-sitosterol for subsequent evaluation of antileishmanial potency against *L. donovani*.

### β-Sitosterol_CCL_ induces intracellular ROS production in promastigotes

**Figure 4:**
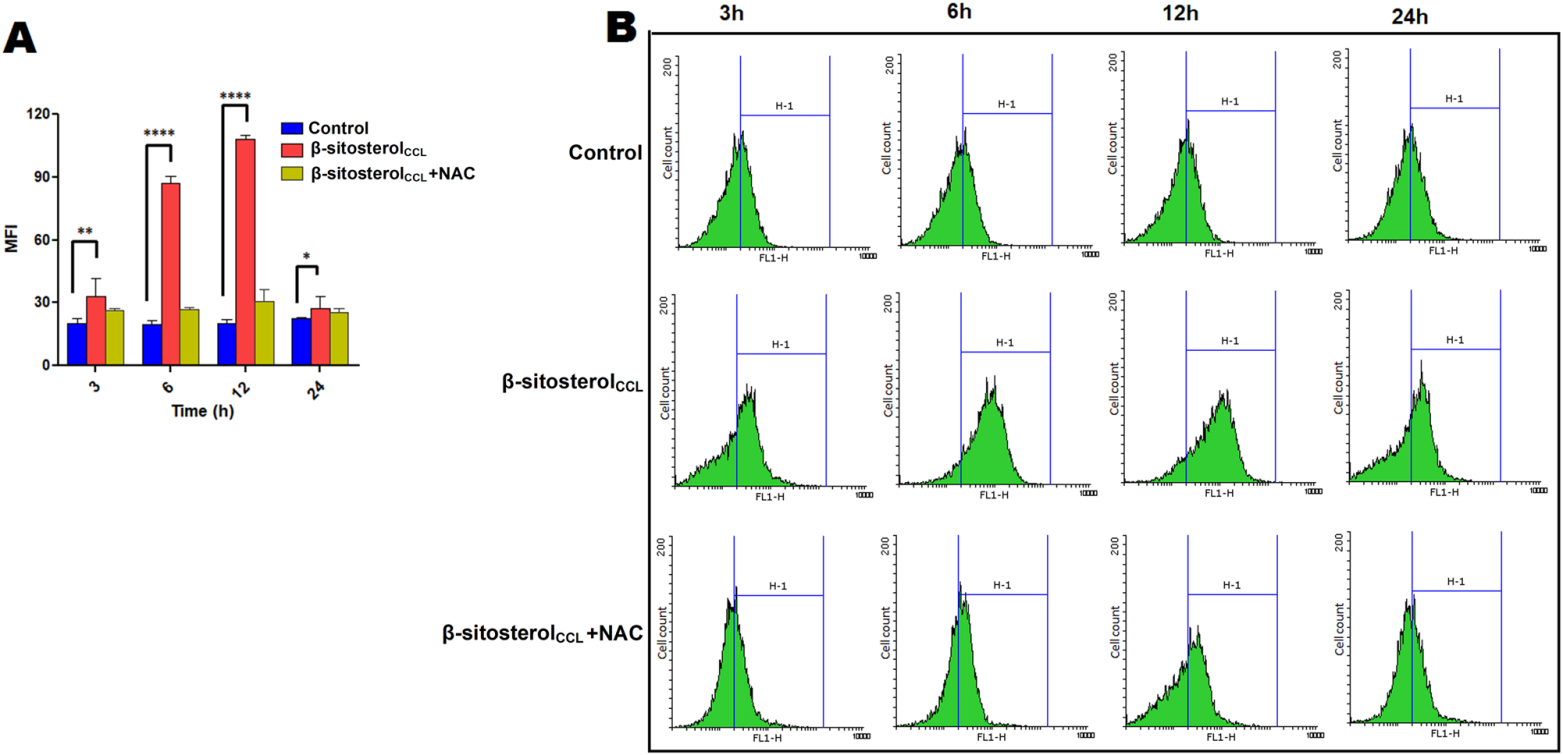
Measurement of intracellular ROS generation in *L. donovani* promastigotes. ROS generation was monitored by flow cytometry using H_2_DCFDA at 3 h, 6 h, 12 h and 24 h. Elevated ROS level in β-sitosterol_CCL_-treated parasites was observed compared to the control, whereas ROS generation in NAC-pre-treated promastigotes was found to be restrained to the level in control cells at each time point. Comparison of ROS generation in untreated, β-sitosterol_CCL_ (IC_50_ dose)-treated and NAC (20 mM)-pre-treated promastigotes at different time points are represented in both the bar graph and histogram. (**A**) The bar graph is representative of three independent experiments, and statistical significance is calculated compared to the untreated control set by using one-way ANOVA with Dunnett's multiple comparison test, where *p < 0.05, **p < 0.01 and ****p < 0.0001 are considered statistically significant. (**B**) Histograms depict the shift of MFI as indicated by the H-1 region. Data were acquired in a BD FACSCalibur flow cytometer and analysed in Flowing software (https://www.flowingsoftware.com), version 2.5.1, Finland.

Intracellular production of ROS is the central factor involved in triggering apoptosis in promastigotes^22^. Hence, we initially monitored the generation of intracellular ROS in β-sitosterol_CCL_-treated *L. donovani* promastigotes by using H_2_DCFDA, a cell-permeable dye. H_2_DCFDA is a nonfluorescent molecule, but once it enters cells, it is ultimately converted to the fluorescent DCF (2′,7′-dichlorofluorescein) by exposure to proper oxidants present within the cells. Therefore, the detected fluorescence of DCF is considered to be an indicator of the intracellular ROS level. Flow cytometry demonstrated that in the early hours of β-sitosterol_CCL_ treatment, there was a gradual time-dependent increase in ROS production for up to 12 h, which triggered oxidative stress in β-sitosterol_CCL_-treated parasites compared to the untreated control. Maximum production of ROS was found at 12 h, after which, ROS generation drastically decreased, reaching close to the level in control cells at 24 h. However, pre-treatment of promastigotes with the ROS quencher NAC (N-acetyl-l-cysteine) restrained the level of ROS generation in β-sitosterol_CCL_-treated cells to the level in control parasites at the same time points. A comparison of the mean fluorescence intensity (MFI) of the treated and control cells is shown in a bar graph (Fig. 4A), and shifting of the fluorescence intensity (FL1-H channel) in β-sitosterol_CCL_-treated parasites compared to the control is also shown by a histogram (Fig. 4B).

### β-Sitosterol_CCL_ alters the morphological structure of promastigotes

**Figure 5:**
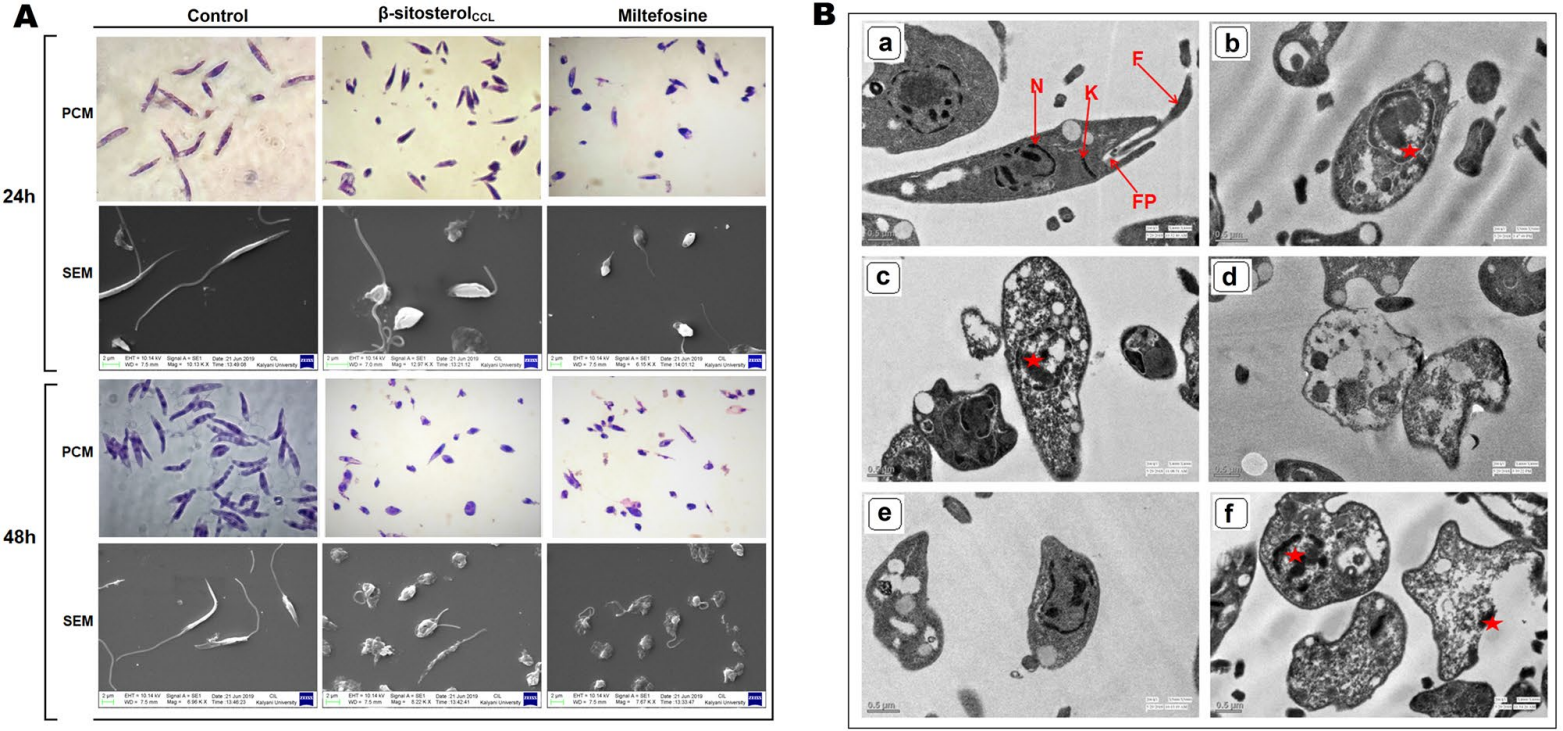
(**A**) Morphological study of *L. donovani* promastigotes. Phase contrast microscopy (PCM) and scanning electron microscopy (SEM) results depicting morphological alterations in β-sitosterol_CCL_ (IC_50_ dose)-treated promastigotes (1 × 10^7^/ml) for 24 h and 48 h compared to control cells. SEM scale bars = 2 μm. (**B**) TEM results showing ultrathin sections of *L. donovani* promastigotes. Promastigotes (1 × 10^7^/ml) were treated with β-sitosterol_CCL_ (IC_50_ dose) for 24 h and 48 h. (**a)** Untreated control, (**b**) β-sitosterol_CCL_-treated promastigotes for 24 h, (**c**,**d**) β-sitosterol_CCL_-treated promastigotes for 48 h, (**e**) miltefosine (10 µm)-treated promastigotes for 24 h, (**f**) miltefosine (10 µm)-treated promastigotes for 48 h. N, nucleus; K, kinetoplast; F, flagellum; FP, flagellar pocket. Scale bar = 0.5 μm. In treated cells, the star (red) indicates the vacuolated nucleus.

Changes in the morphological structure of promastigotes are the key indication of an apoptosis-like mode of cell death^23^. Henceforth, morphological deformities in β-sitosterol_CCL_-treated promastigotes compared to the untreated cells were detected. For comparison, miltefosine (10 µM) was used as a positive control, showing distinct morphological alterations at both 24 h and 48 h. A snapshot captured by phase contrast microscopy (Carl Zeiss, Germany) showed that untreated parasites were structurally organized and elongated in shape with intact flagella after 24 h and 48 h. Similarly, β-sitosterol_CCL_-treated parasites were morphologically atypical, exhibiting features such as roundness with shrinkage and shortening of flagella, compared to the untreated control parasites. This phase contrast microscopic data was also confirmed with SEM imaging of treated and untreated parasites at the same time points (Fig. 5A).

### β-Sitosterol_CCL_ causes ultrastructural changes in promastigotes

Regarding ultrastructural alterations, β-sitosterol_CCL_-treated parasites exhibited notable deformities of internal cellular organelles, such as vacuolated nuclei, distortion of flagellar pockets, and disorganization of mitochondria and kinetoplasts at 24 h and 48 h. However, untreated control parasites displayed normal morphological structures with well-organized intracellular organelles consisting of intact flagellar pockets with flagella, nuclei at central positions and kinetoplasts at the proper location (Fig. 5B). In parallel, miltefosine (10 µm) was used as a positive control for comparison of ultrastructural deformities as observed in the β-sitosterol_CCL_-treated parasites at 24 h as well as 48 h.

### β-Sitosterol_CCL_ triggers lipid body accumulation in promastigotes

**Figure 6:**
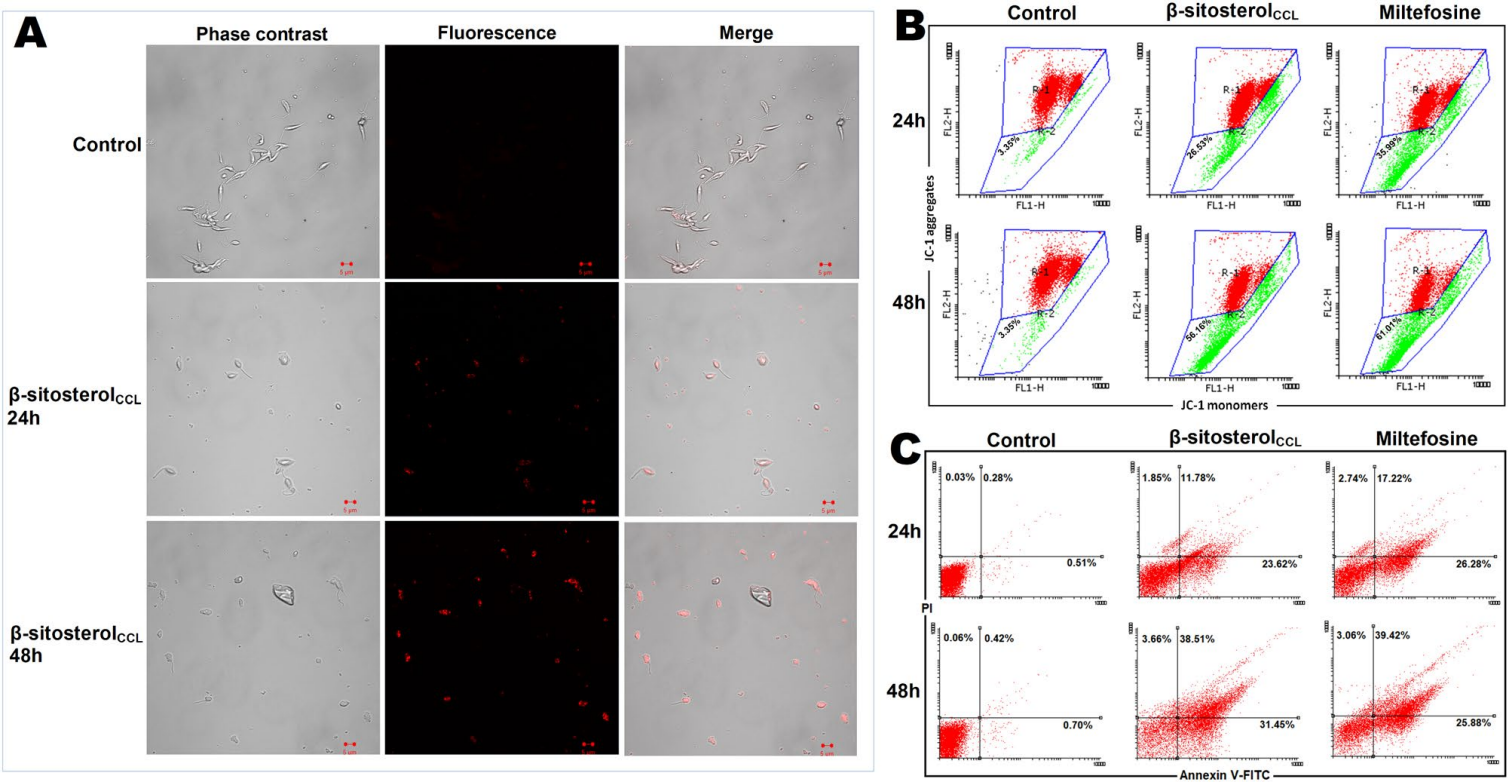
(**A**) Superfluous lipid body detection in *L. donovani* promastigotes. Promastigotes (1 × 10^7^/ml) treated with β-sitosterol_CCL_ for 24 h and 48 h; images were obtained by LSM510-META confocal microscopy after Nile Red staining. Phase contrast, fluorescence, and phase contrast-fluorescence merged images are representative of three independent experiments. (**B**) Flow cytometric determination of mitochondrial membrane depolarization. Promastigotes (1 × 10^7^/ml) treated with β-sitosterol_CCL_ (IC_50_ dose) for 24 h and 48 h followed by JC-1 staining. The fluorescence intensity of JC-1 was measured, and histograms (snapshot) of β-sitosterol_CCL_-treated parasites were compared with control and miltefosine (10 µM)-treated cells for 24 h and 48 h. Red (R-1 region) and green (R-2 region) depict high and low mitochondrial membrane potential, respectively. The snapshots are representative of one of three independent experiments. Data were acquired in a BD FACSCalibur flow cytometer and analysed in Flowing software (https://www.flowingsoftware.com), version 2.5.1, Finland. (**C**) Determination of externalization of phosphatidylserine by flow cytometry. Promastigotes (1 × 10^7^/ml) were treated with β-sitosterol_CCL_ followed by annexin V-FITC and PI staining. Respective dot plots of β-sitosterol_CCL_-treated parasites are represented along with control and miltefosine (10 µM)-treated cells for 24 h and 48 h. Data were acquired in a BD FACSCalibur flow cytometer and analysed in Flowing software (https://www.flowingsoftware.com), version 2.5.1, Finland.

Excess accumulation of intracellular lipid droplets is a key characteristic of cellular stress and triggers apoptosis in parasites^24^. Therefore, while searching for apoptotic events in parasites caused by β-sitosterol_CCL_, we were greatly concerned with evaluating the enhanced accumulation of lipid bodies in treated *L. donovani* promastigotes by using the common fluorescent marker Nile Red, which generally stains intracellular lipid droplets. Upon β-sitosterol_CCL_ treatment for 24 h and 48 h, superfluous lipid droplets were observed to be randomly distributed throughout the cytoplasm of promastigotes, in contrast to the untreated control parasites (Fig. 6A).

### β-Sitosterol_CCL_ causes mitochondrial membrane depolarization in promastigotes

Elevated production of intracellular ROS causes oxidative stress inside promastigotes and subsequently induces mitochondrial membrane depolarization, a major indication of apoptosis^25^. Therefore, the significant effect of β-sitosterol_CCL_ on ROS production made us curious to observe its simultaneous effect on mitochondrial transmembrane potential. Therefore, measurement of mitochondrial membrane depolarization was carried out in promastigotes using the dye JC-1 (5,5′,6,6′-tetrachloro-1,1′,3,3′ tetraethylbenzimidazolcarbocyanine iodide). The dye usually forms aggregates in mitochondria after entering cells. However, in apoptotic cells, aggregated JC-1 is released from mitochondria to the cytoplasm as a monomer. Consequently, aggregated JC-1 at higher potential emits red fluorescence, whereas at lower membrane potentials, JC-1 remains as a monomer within the cytoplasm and emits green fluorescence^26^. Thus, the shift in the cell population towards green fluorescence, as observed by the FACS study, indicates mitochondrial membrane potential depolarization. In the JC-1 assay, FACS data showed that β-sitosterol_CCL_ treatment induced the loss of mitochondrial membrane potential in promastigotes by 26.53% and 56.16% at 24 h and 48 h, respectively (Fig. 6B). Correspondingly, in miltefosine (10 µm)-treated promastigotes, loss of mitochondrial membrane potential was perceived by 35.99% (24 h) and 61.01% (48 h) of the promastigotes.

### β-Sitosterol_CCL_ induces externalization of phosphatidylserine in promastigotes

Externalization of phosphatidylserine to the outer surface of the plasma membrane is the core indication of apoptosis in unicellular eukaryotic cells^27^. Phosphatidylserine exposure is usually analysed by flow cytometry through a dual staining process with annexin V-fluorescein isothiocyanate (annexin V-FITC) and propidium iodide (PI)^27^. In the early stage of cellular apoptosis, when the plasma membrane loses its symmetry, membrane phospholipids are eventually translocated from the inner to outer plasma membrane, and annexin V-FITC rapidly binds with high affinity to the phosphatidylserine exposed to apoptotic cells (early and late). PI selectively binds to DNA in necrotic cells in which membrane integrity is already interrupted, and PI eventually discriminates apoptotic cells from necrotic cells^28^. Thus, the different labelling patterns of annexin V-FITC/PI in the dot plot describe various conditions of cells, which are as follows: annexin V-FITC-negative and PI-negative cells are considered viable, annexin V-FITC-positive and PI-negative cells are considered early apoptotic cells, and annexin V-FITC-positive and PI-positive cells are considered late apoptotic cells, whereas annexin V-FITC-negative and PI-positive cells are considered necrotic cells. Therefore, to determine the apoptotic effect of β-sitosterol_CCL_ in comparison to the effect in untreated control and positive control (miltefosine treated), *L. donovani* promastigotes were double stained with both annexin V-FITC and PI for subsequent flow cytometric analysis. As a result, 26.28% of cells found in the lower-right quadrant were in the early apoptotic phase at 24 h, and 17.22% of cells in the upper-right quadrant were in the late apoptotic stage at the same time point upon miltefosine (10 µM) treatment. After 48 h of miltefosine treatment, 25.88% and 39.42% parasites were detected in the early and late apoptotic stages, respectively, compared to the untreated control parasites (Fig. 6C). Similarly, β-sitosterol_CCL_ treatment for 24 h resulted in 23.62% and 11.78% early and late apoptotic cells, respectively. Increasing the exposure time of β-sitosterol_CCL_ to 48 h increased the amounts of early and late apoptotic cells by 31.45% and 38.51%, respectively (Fig. 6C). Therefore, taken together, these flow cytometry data undoubtedly indicate β-sitosterol_CCL_ to be a potent apoptosis-triggering agent in *L. donovani* promastigotes.

### β-Sitosterol_CCL_ decreases non-protein thiol levels and trypanothione reductase (TryR) activity in promastigotes

**Figure 7:**
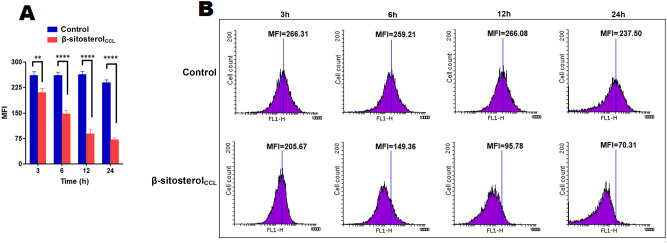
Measurement of intracellular non-protein thiols in promastigotes. Promastigotes (1 × 10^7^/ml) were treated with β-sitosterol_CCL_, and fluorescence intensity was measured after CMFDA staining at various time points, i.e., 3 h, 6 h, 12 h, and 24 h. (**A**) The bar graph depicts the MFI values of three independent experiments, and statistical significance was determined with respect to the control by using a t-test, where **p < 0.01 and ****p < 0.0001 were considered statistically significant. (**B**) Histograms depicting the reduction of MFI in β-sitosterol_CCL_-treated parasites compared to the control at every time point. Data were acquired in a BD FACSCalibur flow cytometer and analysed in Flowing software (https://www.flowingsoftware.com), version 2.5.1, Finland.

Thiols play a key role in protecting parasites against ROS-mediated oxidative stress^29^, and thus, depletion of non-protein thiols could be considered an appropriate focus for drug targeting. In addition, trypanothione reductase (TryR), an indispensable enzyme of kinetoplastid parasites, participates in their unique thiol-redox metabolism^20^. Therefore, the ROS-mediated induction of apoptosis by β-sitosterol_CCL_ made us interested in examining the trypanothione reductase activity and intracellular levels of non-protein thiols. The level of the thiols was monitored flow cytometrically in β-sitosterol_CCL_-treated promastigotes by using the well-known dye 5-chloromethylfluorescein-diacetate (CMFDA). The dye penetrates the cells and usually binds to non-protein thiols and is ultimately converted to a fluorescent thioether^30^. Therefore, the detected fluorescence reflects the level of total non-protein thiols present within the parasites. In β-sitosterol_CCL_-treated promastigotes, a gradual time-dependent decrease in fluorescence intensity was noticed by flow cytometry for up to 24 h compared to the intensity in the control. A comparison of the MFI values of β-sitosterol_CCL_-treated and control parasites is shown in a bar graph (Fig. 7A) and histogram (Fig. 7B).

Since β-sitosterol_CCL_ causes a reduction in the levels of thiols in *Leishmania* promastigotes, TryR inhibition by this component was initially performed in soluble extracts of *L. donovani* promastigotes. The percentage of inhibition of this enzyme in parasite extracts was calculated in terms of the decrease in absorbance at 340 nm, indicating NADPH oxidation. Thus, the optical density itself indicates the consumption of NADPH by TryR. The control lacking any inhibitor was considered as having 100% TryR activity. However, the presence of β-sitosterol_CCL_ (IC_50_ dose) inhibited NADPH consumption by TryR by 52.77 ± 1.04% in parasite extracts compared to the control (Supplementary Fig. S4). To validate the observation, commercial β-sitosterol (Abcam, USA) was also used, which showed a similar inhibitory effect as that of β-sitosterol_CCL_ (Supplementary Fig. S4).

### β-Sitosterol_CCL_ competitively inhibits *Leishmania donovani* trypanothione reductase (*Ld*TryR)

**Figure 8:**
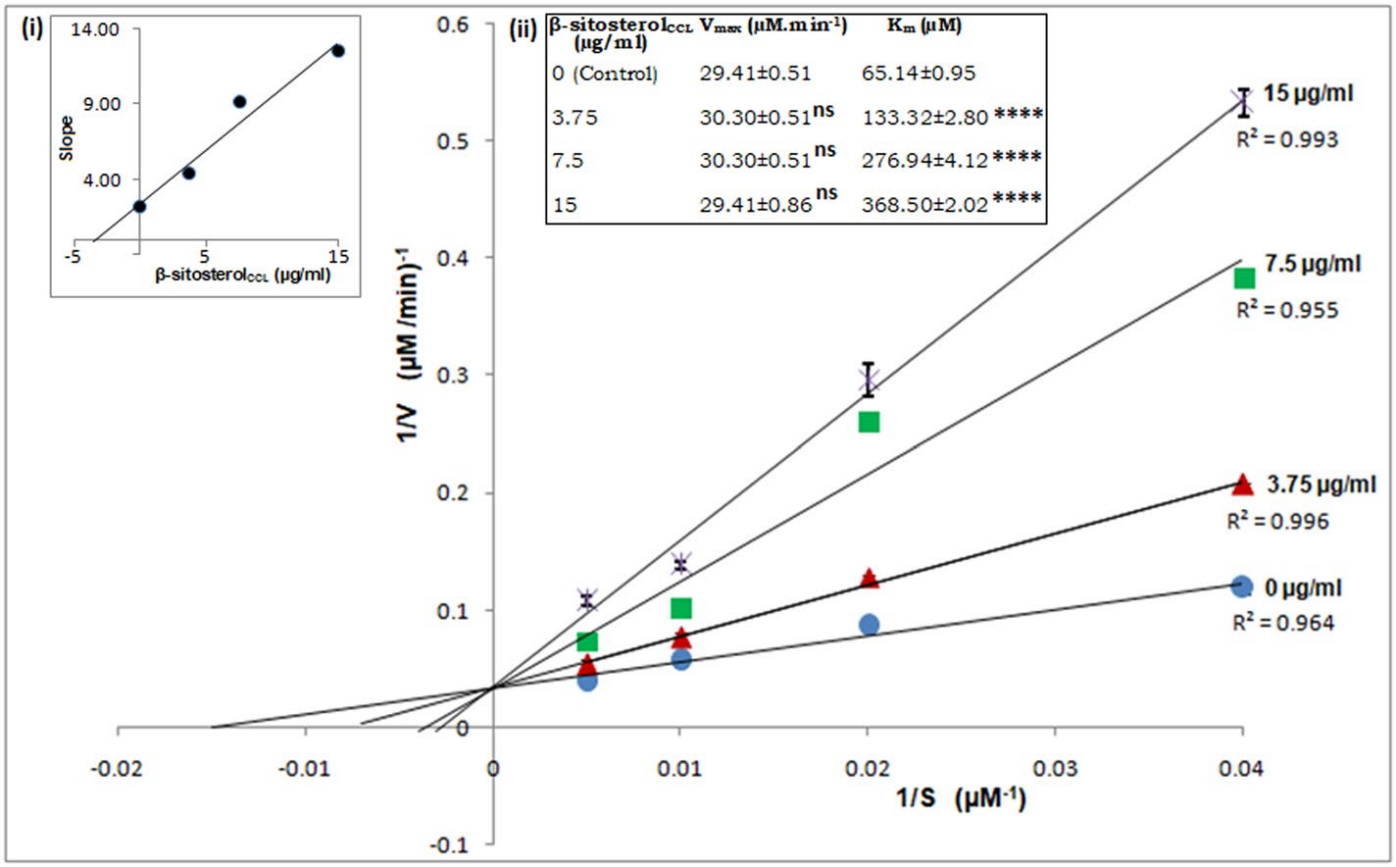
Kinetic analysis of *Ld*TryR inhibition. Lineweaver–Burk plot of *Ld*TryR inhibition by β-sitosterol_CCL_ represents the competitive type. T(S)_2_ was used as the substrate at 25, 50, 100, and 200 µM, and the concentrations of β-sitosterol_CCL_ were 0, 3.75, 7.5 and 15 µg/ml. S, substrate concentration (µM); V, reaction velocity (µmol/min); R^2^, coefficient of determination. The graph in the inset (i) is the secondary replot of the slope of the Lineweaver–Burk plots versus various concentrations of β-sitosterol_CCL,_ and the value of the inhibition constant (K*i*) was calculated as 3.5 µg/ml (8.43 µM). The inset (ii) depicts the kinetic parameters (V_max_ and K_m_) of *Ld*TryR inhibition in the presence of different concentrations of β-sitosterol_CCL_. The statistical significance of the V_max_ and K_m_ values was calculated in comparison to the control by using one-way ANOVA with Dunnett's multiple comparison tests. The values are statistically significant, where **** indicates *p* < 0.0001; “ns” indicates values that are not statistically significant. Herein, values are shown as the mean ± S.D. of three independent studies.

To determine the type of inhibition of *L. donovani* trypanothione reductase (*Ld*TryR) exhibited by β-sitosterol_CCL_, an enzyme kinetics study was performed on recombinant *Ld*TryR. The Lineweaver–Burk plot obtained from the kinetics study showed a gradual increase in apparent K_m_ with increasing β-sitosterol_CCL_ concentration without any effect on Vmax. As a result, the inhibition of *Ld*TryR by β-sitosterol_CCL_ appeared to be competitive, and the inhibition constant (K*i*) of β-sitosterol was found to be 3.5 µg/ml (8.43 µM) (Fig. 8).

### Molecular docking-based binding interactions of β-sitosterol_CCL_ with *Leishmania donovani* trypanothione reductase (*Ld*TryR)

**Figure 9:**
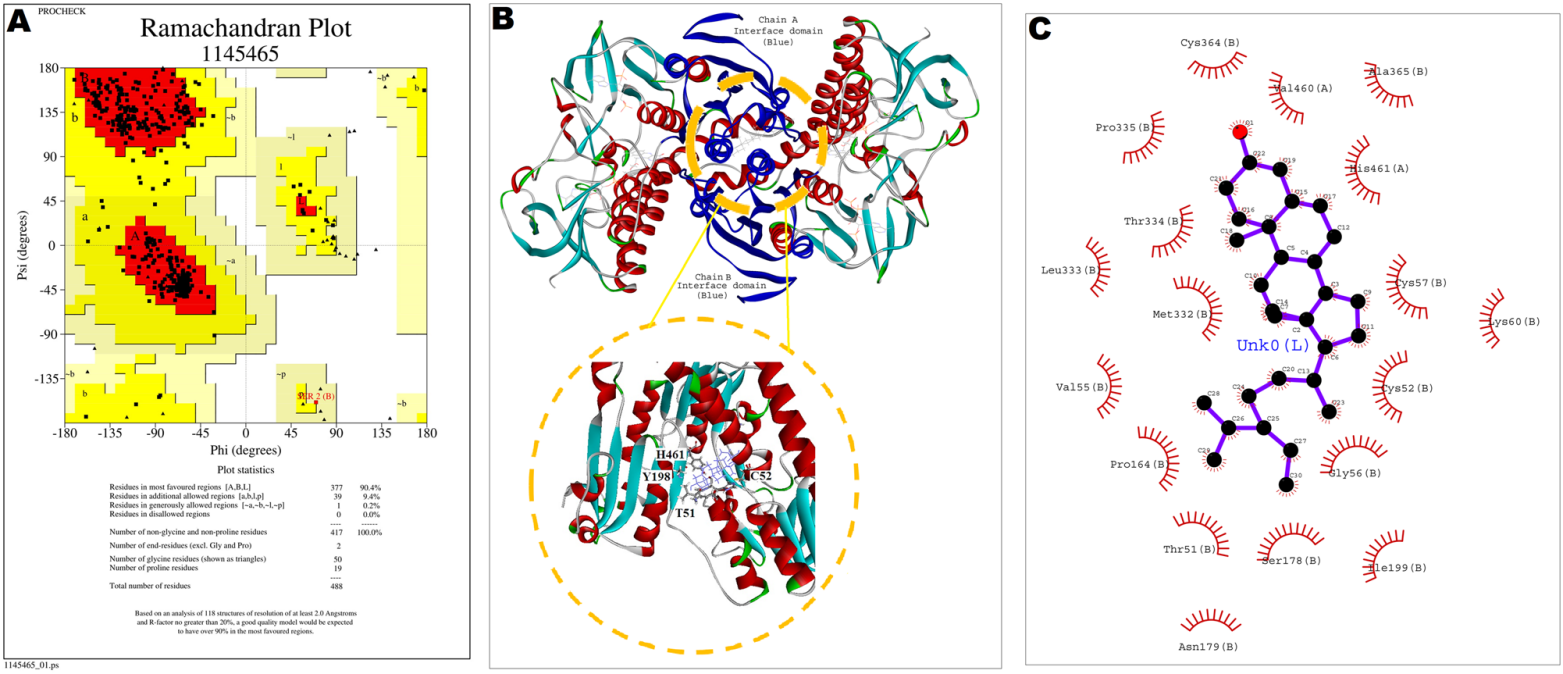
(**A**) Validation of the modelled structure using a Ramachandran plot by PROCHECK analysis. Ramachandran plot showing that 90.4% of amino acid residues are distributed in the allowed region with no amino acids in the disallowed region, indicating good stereochemical fitness of the generated model. (**B**) 3D conformation of the *Ld*TryR-β-sitosterol_CCL_ docked complex, where the circle indicates a close-up view of the binding interactions. (**C**) Ligplot (2D representation) of the docked conformation of *Ld*TryR-β-sitosterol_CCL_ represents adjoining interaction residues of *Ld*TryR denoted by an eyelash mark.

Trypanothione reductase (TryR), an NADPH-dependent enzyme, is unique to kinetoplastid parasites, including *Leishmania*. The key role of TryR in redox metabolism has made it an interesting option for the design of advanced antileishmanial drugs^20,31^. To better understand the binding interactions of the ligand β-sitosterol_CCL_ (*Corchorus capsularis* L. leaf-derived β-sitosterol), molecular docking simulations were performed using the three-dimensional structure of the ligand with *L. donovani* trypanothione reductase (*Ld*TryR). Due to the lack of a crystal structure for *Ld*TryR, homology modelling was performed to generate a three-dimensional model of the protein *Ld*TryR. The homology model of *Ld*TryR was built using the crystallographic structure of TryR from *Leishmania infantum* in complex with TRL156 (PDB Code: 6I7N, chain B), which was used as the template because it shared 98% sequence identity with the *Ld*TryR protein. The modelled structure of *Ld*TryR was superimposed on the crystal structure of the template, and the RMSD of the superimposition was found to be 0.25 Å (Supplementary Fig. S5A). The validation of stereo-chemical qualities of the modelled structure of *Ld*TryR was estimated by the PROCHECK program, and 90.4% of the amino acid residues were in the most favoured region of the Ramachandran plot (Fig. 9A). The structural qualities of the modelled protein were again analysed by Verify3D, and the model passed the criteria of Verify3D; thus, the constructed *Ld*TryR structure was considered a good quality model. Then, the modelled protein (Supplementary Fig. S5B) was docked to the ligand to obtain the three-dimensional (3D) docking conformation with a close-up view of the binding interactions of *Ld*TryR and the ligand, as shown by the circle in Fig. 9B. In this docking analysis, the list of amino acid residues of dimeric *Ld*TryR and their binding energy values with the ligand β-sitosterol_CCL_ are presented in tabular form (Supplementary Table S2), and the total ΔG value of the binding interactions was found to be − 119.4 kcal/mol. The stability of the docked complex was determined for up to 100 ns. Some of the fluctuating amino acid residues identified herein were Pro 42, Asp 84, Asp142, Gly168, Asp 272, Gly 352, Ser 464, and Ser 488 (Supplementary Fig. S5C). The amino acids of *Ld*TryR involved in the binding interactions with the ligand β-sitosterol_CCL_ are shown as two-dimensional (2D) representations (Fig. 9C), which indicate that Val 460 and His 461 were from chain A and Cys 364, Ala 365, Cys 57, Lys 60, Cys 52, Gly 56, Ser 178, Asn 179, Ile 199, Thr 51, Pro 164, Val 55, Met 332, Leu 333, Thr 334, and Pro 335 were from chain B of *Ld*TryR.

## Discussion

Plant-derived natural products possess diverse pharmacological activities and consequently are an attractive resource for the development of advanced chemotherapeutics to treat a wide range of disease conditions, including microbial infections^32^. For the last few years, plant sources have also been reported to be very important in *Leishmania* research^8^. Existing drugs against visceral leishmaniasis exhibit several limitations, such as the emergence of resistance, toxicity and high cost^33,34^. Therefore, in the search for economically feasible antileishmanial agents with better efficacy and low toxicity, plant sources are highly prioritized. In this regard, our previous work on the antileishmanial activity of *C. capsularis* L. leaf extract revealed its efficacy against *L. donovani* promastigotes^9^; therefore, isolation of the natural lead component seems to be significant for exploration of its therapeutic utility in the treatment of VL. Thus, based on the antileishmanial property^9^ and relatively low cytotoxicity of *C. capsularis* L. leaf extract (see Supplementary Method, Results, Fig. S6), the current work was undertaken with the aim of identifying bioactivity-based lead compounds that may play a role in killing parasites. The subsequent bioassay-guided fractionation process resulted in the isolation of a major phytosterol, β-sitosterol, from *Corchorus capsularis* L. leaves (β-sitosterol_CCL_).

In many earlier reports, the medicinal value of plant-derived β-sitosterol has been demonstrated, and the role of β-sitosterol as an antimicrobial agent has also been revealed in the treatment of various infectious diseases^13,14^. β-Sitosterol from a variety of plant sources is applied in the treatment of many parasitic diseases^15,16^. Plant-derived β-sitosterol has also been studied against different forms of leishmaniasis; for example, phytosterols (stigmasterol + β-sitosterol) isolated from *Musa paradisiaca* fruit peel have been tested for antileishmanial properties in vitro by studying growth inhibition of *L. infantum chagasi* promastigotes and amastigotes^19^. Similarly, β-sitosterol isolated with other components from *Sassafras albidum* stem bark was studied against *Leishmania amazonensis*, where the β-sitosterol was found to be least effective against the promastigotes, which was evident from its highest IC_50_ value in comparison to that of the other isolated products^17^. Furthermore, although the leishmanicidal effect of β-sitosterol from *Ifloga spicata* showed apoptosis-type killing of *L. tropica* promastigotes, the detailed mechanism of apoptosis is unclear^18^. Previously, β-sitosterol isolated from *Thalia geniculata* was also tested against *L. donovani* amastigotes (strain MHOM/ET/67/L82), but no significant activity of β-sitosterol was documented^16^. Therefore, despite the many studies on the effect of plant-derived β-sitosterol on various forms of leishmaniasis, the mechanisms by which the compound kills the parasites remain unclear. On the basis of previous reports, it could be suggested that although the effect of different plant-derived β-sitosterols has been examined on various *Leishmania* spp., the effectiveness varies among different *Leishmania* species and strains^16–19,35^. The discrepancy in the efficacy of drugs depends on the phenotypic variability of different species or strains, clinical appearances and geographical origin^36,37^^.^ Certain antileishmanial drugs have also been reported to exhibit species- and strain-specific efficiency^37–40^^.^ Additionally, various plant-derived secondary metabolites have been shown to exhibit target-specific activity against parasites^15^. Therefore, considering these aspects, the present investigation of *Corchorus capsularis *L. leaf-derived β-sitosterol (β-sitosterol_CCL_) against the Indian strain of *L. donovani* promastigotes [(MHOM/IN/1983/AG83)] seems very reasonable and unique.

As β-sitosterol is available commercially, during our investigation of the antileishmanial properties of β-sitosterol_CCL_, it was also of interest to evaluate the efficacy of commercial β-sitosterol on *L. donovani* promastigotes. Therefore, commercial β-sitosterol was initially compared herein with β-sitosterol_CCL_ in terms of its antileishmanial properties and cytotoxicity. Although both β-sitosterol_CCL_ and commercial β-sitosterol exhibited potent antileishmanial activity, β-sitosterol_CCL_ was less toxic than commercial β-sitosterol. Similar observations have also been reported previously, showing that plant extracts or plant-derived components are safer and more efficient than any synthetic drugs against parasitic diseases^18^. As a result, the current investigation on the natural lead component β-sitosterol from the commonly available edible plant *C. capsularis* L. focused on β-sitosterol_CCL_ as a novel agent for elucidating the cell death mechanism in *L. donovani*.

In the present study, β-sitosterol_CCL_ initially led to the appearance of major features of apoptosis, such as the formation of intracellular ROS and, subsequently, an atypical morphology of *L. donovani* promastigotes with alterations in internal organelles. Similar morphological and ultrastructural changes were observed during apoptotic death of *L. donovani* promastigotes treated with clerodane diterpene (K-09) obtained from *Polyalthia longifolia* leaves^24^. Lipid droplets are very special, dynamic and complex organelles that play a key role in regulating lipid metabolism in unicellular protozoan parasites^41^. Surplus cytoplasmic accumulation of these lipid droplets is also regarded as a symbolic feature of apoptotic cells^42^. A report suggests that the dibenzylideneacetone A3K2A3 exerts leishmanicidal activity through the excess accumulation of lipid bodies within the parasites^43^. An elevated number of lipid droplets upon clerodane diterpene K-09 exposure was also noted in *L. donovani* parasites^24^. Generally, impeding lipid metabolism causes excess production of lipid precursors that accumulate in the form of lipid bodies inside cells and provoke cell death^24,42^. Correspondingly, in the current study, the antiparasitic nature of β-sitosterol_CCL_ was also observed as enhanced accumulation of lipid bodies in β-sitosterol_CCL_-treated parasites, which ultimately led to parasite killing due to probable alteration of lipid metabolism.

*Leishmania* parasites possess a single mitochondrion that plays an essential role in the survival of parasites by maintaining homeostasis; thus, loss of mitochondrial membrane potential could be considered a very striking characteristic of cell death by means of apoptosis^22,23^. Interestingly, an appreciable time-dependent decrease in the mitochondrial membrane potential was noticed in β-sitosterol_CCL_-treated parasites and consequently provided an excellent indication of apoptosis. Subsequently, depolarization of the mitochondrial membrane potential allowed us to explore the apoptosis promotion capability of this β-sitosterol_CCL_ in *L. donovani* promastigotes. Apoptosis is a common physiological phenomenon that leads cells towards death^27^. In higher eukaryotic unicellular organisms, apoptosis is represented by externalization of phosphatidylserine from the inner leaflet to the outer surface of the plasma membrane. Interestingly, increased exposure of phosphatidylserine was similarly noticed in *L. donovani* promastigotes after β-sitosterol_CCL_ treatment, which further confirms the mode of parasite killing exhibited by this natural lead component.

Furthermore, the ROS-mediated apoptosis-like programmed cell death events in promastigotes caused by β-sitosterol_CCL_ exposure inspired us to explore the effect of the compound on non-protein thiols, which play an important role in protecting parasites under oxidative stress condition^29,30^. Fascinatingly, in the current study, significant depletion of non-protein thiol levels was also noticed in *L. donovani* promastigotes upon β-sitosterol_CCL_ treatment. Thus, β-sitosterol_CCL_ causes redox imbalance situations with increased ROS production and decreased antioxidant-like thiol levels in *L. donovani* promastigotes followed by apoptosis. Moreover, the viability and infectivity of *Leishmania* parasites generally depend on some key enzymes, the major enzyme among which is a redox-maintaining enzyme, trypanothione reductase (TryR). In general, the redox balance in *Leishmania* is regulated by TryR, which provokes a cascade of events via the reduction of trypanothione disulphide [T(S)_2_] to the dithiol form [T(SH)_2_] by neutralization of ROS^20^. Thus, the antileishmanial function of β-sitosterol_CCL_ targeting the enzyme TryR appears very imperative and relevant in the current study. Consequently, an enzymatic assay was performed, which showed that β-sitosterol_CCL_ significantly inhibits TryR activity in soluble extracts of *L. donovani* promastigotes. Therefore, a subsequent enzyme kinetics study on recombinant *Ld*TryR was performed with β-sitosterol_CCL_ and clearly demonstrated that β-sitosterol_CCL_ is an efficient competitive inhibitor of *Ld*TryR. This further confirms that β-sitosterol_CCL_ acts as a potent antileishmanial agent by competitively inhibiting the enzyme *Ld*TryR. Although many synthetic compounds are also reported to inhibit the parasitic trypanothione reductase enzyme in a competitive manner^21,44,45^, the inhibition of *Ld*TryR by β-sitosterol obtained from *C. capsularis* L. leaves is the first report of an efficient antileishmanial agent. Therefore, the significant inhibitory effect of β-sitosterol_CCL_ against *Ld*TryR led us to check the binding affinity of the compound with the enzyme with the help of molecular docking simulation.

Molecular modelling, a computer-assisted tool, has emerged as an attractive platform for better understanding drug design and is widely used to predict the preferred binding orientation of pharmacologically active molecules (ligands) with biological macromolecules^46^. Trypanothione reductase (TryR) is an NADPH-dependent homodimer that comprises the cofactor FAD bound to each subunit and helps in electron transfer from NADPH to oxidized trypanothione through the prosthetic group, FAD and a redox-active cysteine disulphide residue^20,47^. Previously, many phytocompounds and synthetic compounds have been reported to dock with TryR from different *Leishmania* spp.^18,20,21^. Docking studies have been performed between TryR from *L. infantum* (PDB ID 4APN) and β-sitosterol^18^. However, the efficiency of plant-derived β-sitosterols in the binding of *L. donovani* trypanothione reductase has yet to be studied. Hence, we used molecular docking to investigate the binding capacity of β-sitosterol (β-sitosterol_CCL_) with trypanothione reductase of *L. donovani*. The X-ray crystallographic structure of *L. donovani* trypanothione reductase (*Ld*TryR) is not well documented^21^. Therefore, we employed homology modelling to generate the three-dimensional structure of *Ld*TryR using the X-ray crystallographic structure of TryR from *Leishmania infantum* in complex with TRL156 (PDB Code: 6I7N, chain B) as a template. The sequence identity between *Ld*TryR and the template was 98%. Then, we made a structural comparison between the modelled structure of *Ld*TryR with the template 6I7N, chain B by structurally aligning their C-α backbone atoms. The root mean squared deviation (RMSD) between the backbones of the proteins was found to be 0.25 Å, which indicates a significant structural similarity between the modelled *Ld*TryR and the template 6I7N, chain B. Ultimately, the modelled structure of *Ld*TryR was used to dock with the ligand β-sitosterol (β-sitosterol_CCL_) in the presence of FAD (cofactor) and NADPH; the stability of the molecular interaction was also established from the ΔG value. Next, we tried to compare the distribution of the active site amino acid residues of *Ld*TryR with the same proteins from other members of the *Leishmania* and *Trypanosoma* families. For that purpose, we used the amino acid residues of the following sets of proteins:

- *LiTryR* from *Leishmania infantum* TryR.
- *Tc*TryR from *Trypanosoma cruzi* TryR.
- *Lb*TryR from *Leishmania braziliensis* TryR.

We compared the amino acid sequences of the above mentioned proteins by a multiple sequence alignment method and identified the conserved active site amino acid residues in the proteins (Supplementary Fig. S7). These residues are conserved in all species. In our model, all these residues were also found to be involved in binding interactions, which take place in and around the active sites. Therefore, the in silico interaction study again validates the observations of the inhibitory activity of β-sitosterol_CCL_ targeting TyrR of *L. donovani* promastigotes.

**Figure 10:**
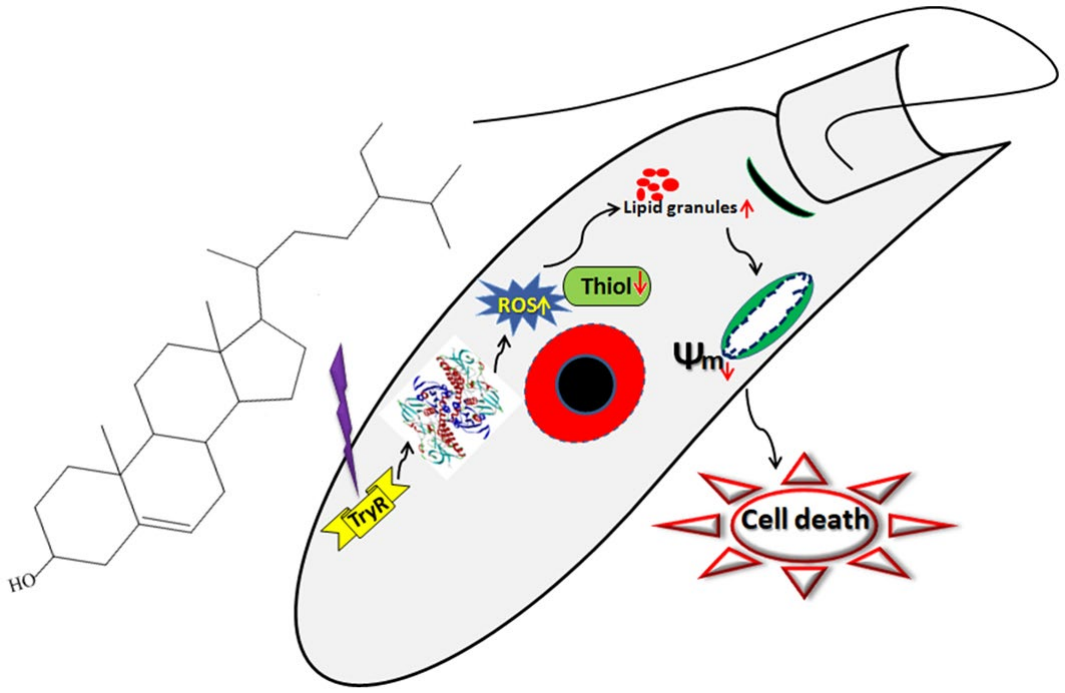
Proposed model of *C. capsularis* L. leaf-derived β-sitosterol (β-sitosterol_CCL_)-mediated inhibition of *Ld*TryR in parasite killing.

Overall, the antileishmanial activity of β-sitosterol_CCL_ against *L. donovani* promastigotes exhibits very specific apoptotic features by targeting *Ld*TryR, as depicted in the proposed model (Fig. 10). Thus, the present work might provide an efficient way to use *Corchorus capsularis* L. leaf-derived β-sitosterol (β-sitosterol_CCL_) as an alternative novel therapeutic agent against visceral leishmaniasis in the future.

## Conclusion

The present investigation introduces *Corchorus capsularis* L. leaf-derived β-sitosterol (β-sitosterol_CCL_) as a novel antileishmanial agent that competitively inhibits *Leishmania donovani* trypanothione reductase. Overall, the antiparasitic efficiency and the ability of this phytosterol to block one of the most crucial parasitic enzymes emphasize its proficiency as a strong candidate for treatment of visceral leishmaniasis.

## Materials and methods

### Cell culture

The Indian strain of *Leishmania donovani* (MHOM/IN/1983/AG83) promastigotes was cultured at 22 °C in medium 199 (Sigma-Aldrich) with 100 U/ml penicillin (Gibco) and 100 mg/ml streptomycin (Gibco) with 10% (v/v) heat-inactivated foetal calf serum (Gibco). The murine macrophage cell line RAW 264.7 was also maintained in RPMI 1640 at 37 °C in 5% CO_2_^48^.

### Bioassay-guided fractionation and isolation of compounds

Chloroform extract of *Corchorus capsularis* L. leaves was subjected to silica gel (Merck, pore size 60–120) column chromatography and eluted with different step gradients of hexane–ethyl acetate by increasing the proportion of ethyl acetate. A total of 441 fractions of 50 ml each were collected. Based on thin layer chromatography (TLC) (Merck) profiles, fractions were pooled to obtained thirteen major fractions (F1 to F13). These fractions (F1-F13) were evaporated to dryness on rotary evaporator (Buchi, Switzerland).

The fractions (F1-F13) were screened against *L. donovani* promastigotes, testing for viability, by the MTT assay^9^. Log-phase promastigotes (1 × 10^7^/ml) were seeded in 96-well plates (BD falcon) and treated separately with these fractions (0–200 µg/ml) for 48 h. Next, MTT was added, and the plate was incubated for 6 h at 37 °C. Then, viable cells were estimated by conversion of MTT to formazan at 570 nm in an iMark Microplate Reader (Bio-Rad). The IC_50_ value of each fraction was calculated by GraphPad Prism software (version 5).

### Characterization of ***Corchorus capsularis*** L. leaf-derived β-sitosterol (β-sitosterol_CCL_)

FTIR analysis of the isolated lead compound was carried out to identify the existing functional groups. A thin disc of the sample was prepared by using a KBr pellet, and the spectral data were recorded by FTIR spectrometry (Perkin Elmer)^49^. ^1^H and ^13^C NMR spectra were scanned with a Bruker Avance spectrometer at 400 MHz and 100 MHz, respectively, by using CDCl_3_ as the solvent system^49^. GC–MS analysis was performed with a gas chromatograph (Agilent Technologies 7980A) equipped with a mass spectrometric system (7000, GC/MS triple quad). An HP-5MS column (30 m length, 0.25 mm I.D., film thickness 0.25 μm) was employed^50^. Agilent Mass Hunter software (Version B.50.00) was used for instrument control and data analysis.

### Surface morphology analysis of β-sitosterol_CCL_

The compound was dried under vacuum for scanning electron microscopy (SEM) imaging. Images of the surface morphology of the compound were then obtained by SEM (ZEISS EVO LS 10).

### Antipromastigote activity and cytotoxicity assay of β-sitosterol_CCL_ and commercial β-sitosterol

To check the inhibitory effect of commercial β-sitosterol on the growth of *L. donovani* promastigotes, an MTT assay was performed as described previously^9^. Promastigotes (1 × 10^7^/well) were treated individually with β-sitosterol_CCL_ and commercial β-sitosterol (Abcam, USA) (0–200 µg/ml) for 48 h. The IC_50_ value was determined from a graphical representation (GraphPad Prism software, version 5).

Similarly, cytotoxicity was tested on RAW 264.7 macrophages (1 × 10^5^/ml) by an MTT assay with β-sitosterol_CCL_ and commercial β-sitosterol. Macrophages were treated with both the β-sitosterols at a dose of 0 to 200 µg/ml for 48 h. The percentage of host cells affected by the compounds was calculated through graphical exploitation by using GraphPad Prism software (version 5).

### Measurement of ROS in β-sitosterol_CCL_-treated promastigotes

*Leishmania donovani* promastigotes (1 × 10^7^/ml) were treated with β-sitosterol_CCL_ (IC_50_ dose) for 3 h, 6 h, 12 h and 24 h. Then, the cells were washed with PBS and incubated with the cell-permeable probe 2,7-dichlorodihydrofluorescein diacetate (H_2_DCFDA)^51^ for 30 min. One set of cells was pre-treated with 20 mM ROS quencher (NAC; Sigma–Aldrich) at the same time points. The intensity of the fluorescence signal was then acquired with a BD FACSCalibur flow cytometer and analysed by Flowing software (version 2.5.1, Finland)^52^.

### Parasite morphology analysis of β-sitosterol_CCL_-treated promastigotes

Promastigotes were treated with β-sitosterol_CCL_ (IC_50_ dose) for 24 h and 48 h. Then, treated and untreated cells were fixed in methanol and stained with Giemsa (Sigma-Aldrich). Subsequently, images were obtained under a Meiji (ML 2955) light microscope (100× objective). For scanning electron microscopy (SEM), fixation of cells was performed in 2.5% glutaraldehyde and 2% paraformaldehyde for 3 h at room temperature, and the cells were left overnight at 4 °C^53^. Then, the samples were dehydrated with an increasing gradient of ethanol washing solution and imaged under a scanning electron microscope (ZEISS EVO LS 10). Herein, miltefosine (10 µm) was used as a positive control.

### Ultrastructural study of β-sitosterol_CCL_-treated promastigotes

Promastigotes were treated with β-sitosterol_CCL_ (IC_50_ dose) for 24 h and 48 h. Cells were then fixed with 2.5% glutaraldehyde and 2% paraformaldehyde in sodium cacodylate buffer (pH 7.2). Then, samples were prepared for TEM, and ultrastructural imaging was performed using a Tecnai G2 20 S-Twin transmission electron microscope at SAIF, AIIMS, New Delhi^54^.

### Detection of lipid droplet accumulation in β-sitosterol_CCL-_treated promastigotes

Excess accumulation of lipid bodies within parasites was detected by using Nile Red dye^55^. Briefly, promastigotes (1 × 10^7^ cells/ml) were treated with β-sitosterol_CCL_ (IC_50_ dose) at 24 h and 48 h. Cells were incubated with 10 μg/ml Nile Red for 30 min in the dark and then fixed with 4% paraformaldehyde in phosphate buffer (0.1 M, pH 7.2). Parasites were then imaged under LSM510-META confocal microscopy (Carl Zeiss, Germany)^56^ at 480 nm for excitation and 530 nm for emission.

### Determination of mitochondrial membrane potential in β-sitosterol_CCL_-treated promastigotes

The mitochondrial membrane potential in treated and untreated parasites was assessed flow cytometrically by using the cell-permeable dye JC-1^22^. Promastigotes (1 × 10^7^ cells/ml) were treated with β-sitosterol_CCL_ for 24 h and 48 h. After washing with PBS, the cells were incubated with JC-1 (BD Biosciences) for 15 min at 37 °C according to the manufacturer’s protocol. Then, data were acquired in a BD FACSCalibur flow cytometer and analysed in Flowing software (version 2.5.1, Finland).

### Detection of phosphatidylserine externalization in β-sitosterol_CCL_-treated promastigotes

Promastigotes (1 × 10^7^ cells/ml) were treated with an IC_50_ dose of β-sitosterol_CCL_ for 24 h and 48 h. The washed cell pellet was resuspended in 1× binding buffer. Subsequently, the samples were incubated in the dark for 20 min after the addition of annexin V-FITC and PI^22^. Data acquisition and analysis were performed in a BD FACSCalibur flow cytometer and Flowing software (version 2.5.1, Finland), respectively.

### Measurement of non-protein thiols in β-sitosterol_CCL_-treated promastigotes

Promastigotes (1 × 10^7^ cells/ml) were treated with an IC_50_ dose of β-sitosterol_CCL_ at 3 h, 6 h, 12 h and 24 h. After treatment, the cells were incubated with 10 μM CMFDA (Molecular Probes) in the dark for 30 min^30^. The samples were acquired in a BD FACSCalibur flow cytometer and analysed in Flowing software (version 2.5.1, Finland).

#### **Trypanothione reductase assay**

Soluble extract of *L. donovani* promastigotes was obtained by resuspending the washed pellet in buffer containing 40 mM HEPES (pH 7.4) and 1 mM EDTA^57^. The cell suspension was then lysed in a Dounce homogenizer and centrifuged at 12,000×*g* for 15 min. The supernatant collected was considered the soluble fraction containing trypanothione reductase. Then, the assay was performed by incubating the soluble protein fraction (1 mg/ml) for 10 min with β-sitosterol_CCL_ (IC_50_ dose). Then, the reaction was initiated by the addition of NADPH (0.1 mM) in HEPES (40 mM) and EDTA 1 (mM) plus 100 mM substrate (trypanothione disulphide, Sigma-Aldrich). Herein, commercial β-sitosterol (Abcam, USA) was also tested in parallel, and a positive control set was made by incubation with clomipramine (10 µM), a known TryR inhibitor^31^. TryR activity was measured in a spectrophotometer (Hitachi, Japan) by measuring NADPH consumption at 340 nm. The percentage of inhibition was eventually calculated based on a decrease in optical density^20,57^.

### Enzyme kinetics study of the effect of β-sitosterol_CCL_ on recombinant *Leishmania donovani* trypanothione reductase (*Ld*TryR)

Recombinant *L. donovani* trypanothione reductase (*Ld*TryR)^47^ was kindly provided by Dr. Neena Goyal, The CSIR-Central Drug Research Institute, Lucknow, India. An enzyme inhibition assay was performed spectrophotometrically^58^. The enzyme inhibition kinetics were determined in an assay mixture (40 mM HEPES, 1 mM EDTA at pH 7.5) containing *Ld*TryR and the substrate trypanothione disulphide T(S)_2_ (Sigma-Aldrich) at varying concentrations (25, 50, 100 and 200 µM), where β-sitosterol_CCL_ as an inhibitor was added at concentrations of 0, 3.75, 7.5, and 15 µg/ml. The reactions were monitored by the addition of 100 µM NADPH (Sigma-Aldrich), and changes in absorbance were measured at 340 nm^44^. In the presence of β-sitosterol_CCL_, the mode of inhibition was detected by a Lineweaver–Burk plot, and the value of the inhibition constant (K*i*) was calculated.

### Molecular docking simulation

The three-dimensional coordinates of the atoms of the ligand β-sitosterol were obtained from Pubchem^59^. The amino acid sequence of the protein *Ld*TryR from *L. donovani* was retrieved from UniProt with the accession number P39050. We used homology modelling to build the three-dimensional structure of *Ld*TryR using 6I7N and chain B as templates. The stereo-chemical qualities of the modelled structure were validated through a Ramachandran plot by using the PROCHECK program. The structural qualities of the modelled protein were again analysed by Verify3D. A molecular docking study between the modelled structure of *Ld*TryR and ligand (β-sitosterol_CCL_) was performed using the tool GOLD, and the best docking poses were chosen as per the GOLD scores^60^.

### Statistical analysis

Statistical analysis was performed in the GraphPad Prism program (version 5.0 for Windows; Fay Avenue, La Jolla, CA, USA). The data are representative of three separate experiments and expressed as the mean ± standard error (S.E.)/standard deviation (S.D.) as stated in the figure legends. The significance of differences was calculated by using Student’s t-test (two-group comparison) and one-way ANOVA with Dunnett's multiple comparison test (multiple-group comparison), where *p < 0.05, **p < 0.01, ***p < 0.001, and ****p < 0.0001 were considered statistically significant.

## Supplementary information

Supplementary Information.
