# Supplementary material for: Bioassay-based Corchorus capsularis L. leaf-derived β-sitosterol exerts antileishmanial effects against Leishmania donovani by targeting trypanothione reductase
Source: Sci Rep. 2020 Nov 24;10:20440. doi: 10.1038/s41598-020-77066-2 (PMC7686382; doi:10.1038/s41598-020-77066-2)
Supplement: Supplementary file 1 — Supplementary Information. [file 41598_2020_77066_MOESM1_ESM.pdf]

# Supplementary Information

## Bioassay-based *Corchorus capsularis* L. leaf-derived β-sitosterol exerts antileishmanial effects against *Leishmania donovani* by targeting trypanothione reductase

Pijush Kanti Pramanik[1], Sajal Chakraborti[1], Angshuman Bagchi[1], Tapati Chakraborti[1*]

[1]Department of Biochemistry and Biophysics, University of Kalyani, Kalyani 741235, West Bengal, India.

*Address for correspondence:
Dr. Tapati Chakraborti, PhD
Professor,
Department of Biochemistry and Biophysics
University of Kalyani, Kalyani 741235
West Bengal, India
E-mail: tcbiochem@gmail.com

**Table of Contents**

1. Supplementary Result
2. Supplementary Method
3. Supplementary Figures
4. Supplementary Tables
5. Supplementary References

## 1. Supplementary Result

**Assessment of cytotoxicity of *Corchorus capsularis* L. leaf extract.** Cytotoxic effect of chloroform extract of *Corchorus capsularis* L. leaf was investigated against host macrophages by MTT assay method and dose response graph demonstrated that only 11.29±0.37% cells with reference to control were affected with the highest dose (1000 μg/ml) of the leaf extract even after 48 h of treatment (Supplementary Fig. S6). Furthermore, the particular dose at which 50% of promastigotes is deceased as shown in our previous report[1], that dose was safe on host macrophages.

## 2. Supplementary Method

**Cytotoxicity assay.** In order to check the toxic effect of chloroform extract of *Corchorus capsularis* L. leaf on host murine macrophages, RAW 264.7 macrophages cell line was maintained in RPMI 1640 supplemented with 10% FCS (Gibco) in addition of 100 U/ml penicillin and 100 mg/ml streptomycin at 37$^{o}$C in 5% $CO_2$ atmosphere[2]. Afterwards, viability of macrophages was assessed by previously described trypan blue (Sigma-Aldrich) exclusion method[3]. Then, RAW 264.7 macrophages ($1\times10^{5}$/ml) were adhered into a 96-well plate (BD falcon) and treated with the extract at the concentration dose ranging from 0 to 1000 μg/ml for 48 h. After 48 h of treatment MTT was added then amount of formazan produced which is directly proportional to the number of metabolically active cells was finally measured at 570 nm in iMark Microplate Reader (Bio-Rad).

## 3. Supplementary Figures

**Figure S1.**

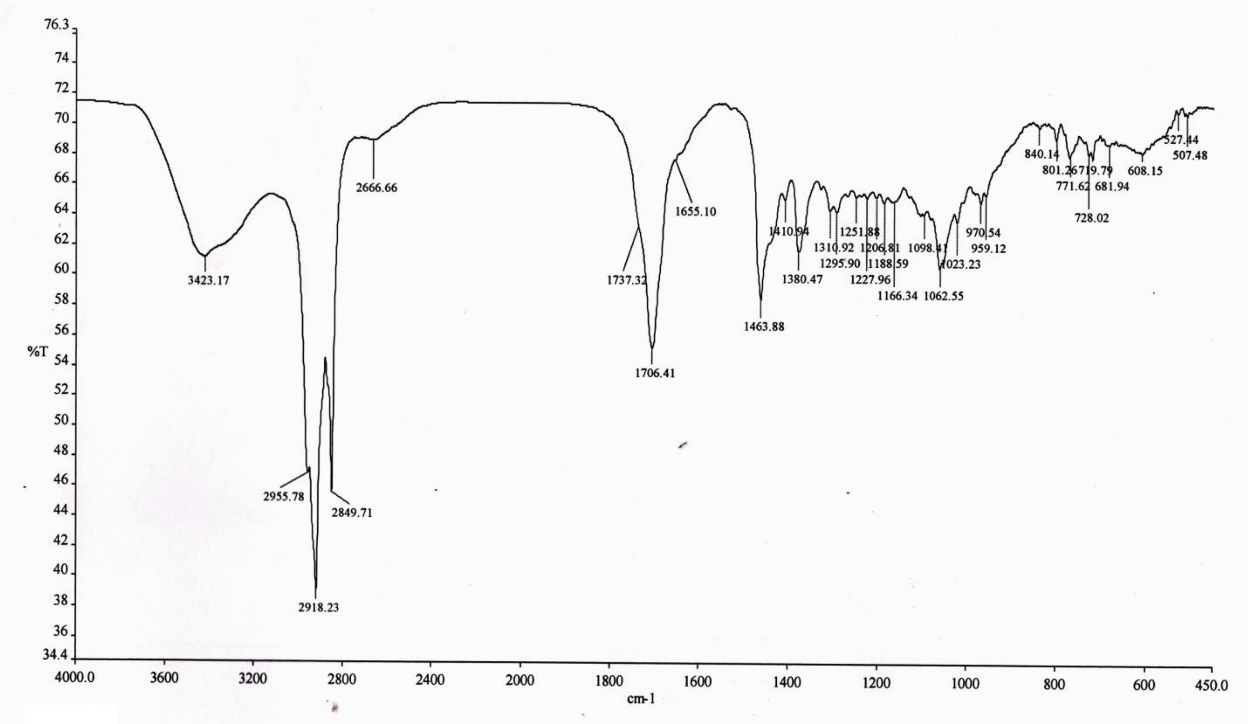


**Figure S1.** FTIR absorption spectrum of *Corchorus capsularis* L. leaf derived β-sitosterol (β-sitosterol$_{CCL}$). The Spectrum was recorded in Perkin Elmer FTIR spectroscopy ranging from 450 to 4000 cm$^{-1}$.

**Figure S2.**

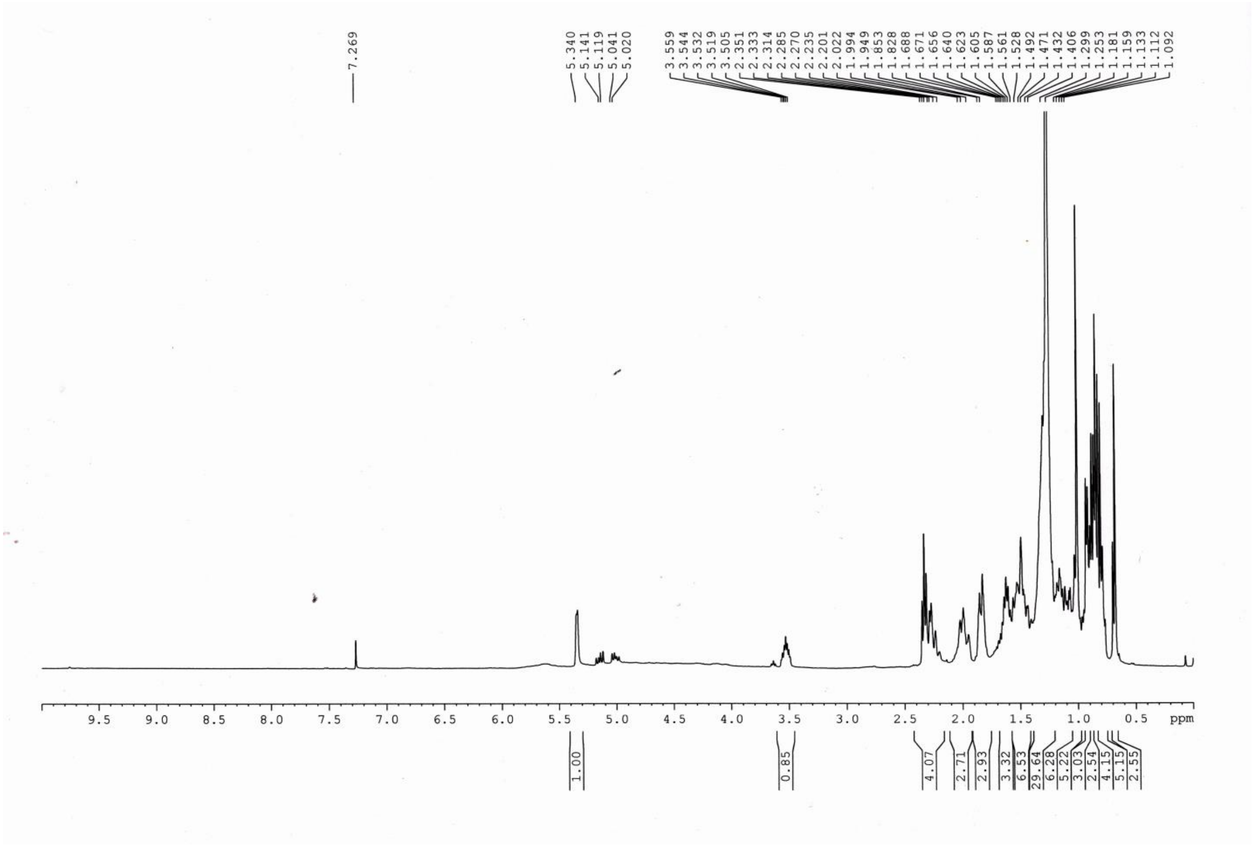


**Figure S2.** $^{1}H$ NMR spectrum of *Corchorus capsularis* L. leaf derived β-sitosterol (β-sitosterol$_{CCL}$). The NMR spectrum was recorded in a Bruker Avance spectrometer at 400 MHz by using $CDCl_3$ as solvent system.

**Figure S3.**

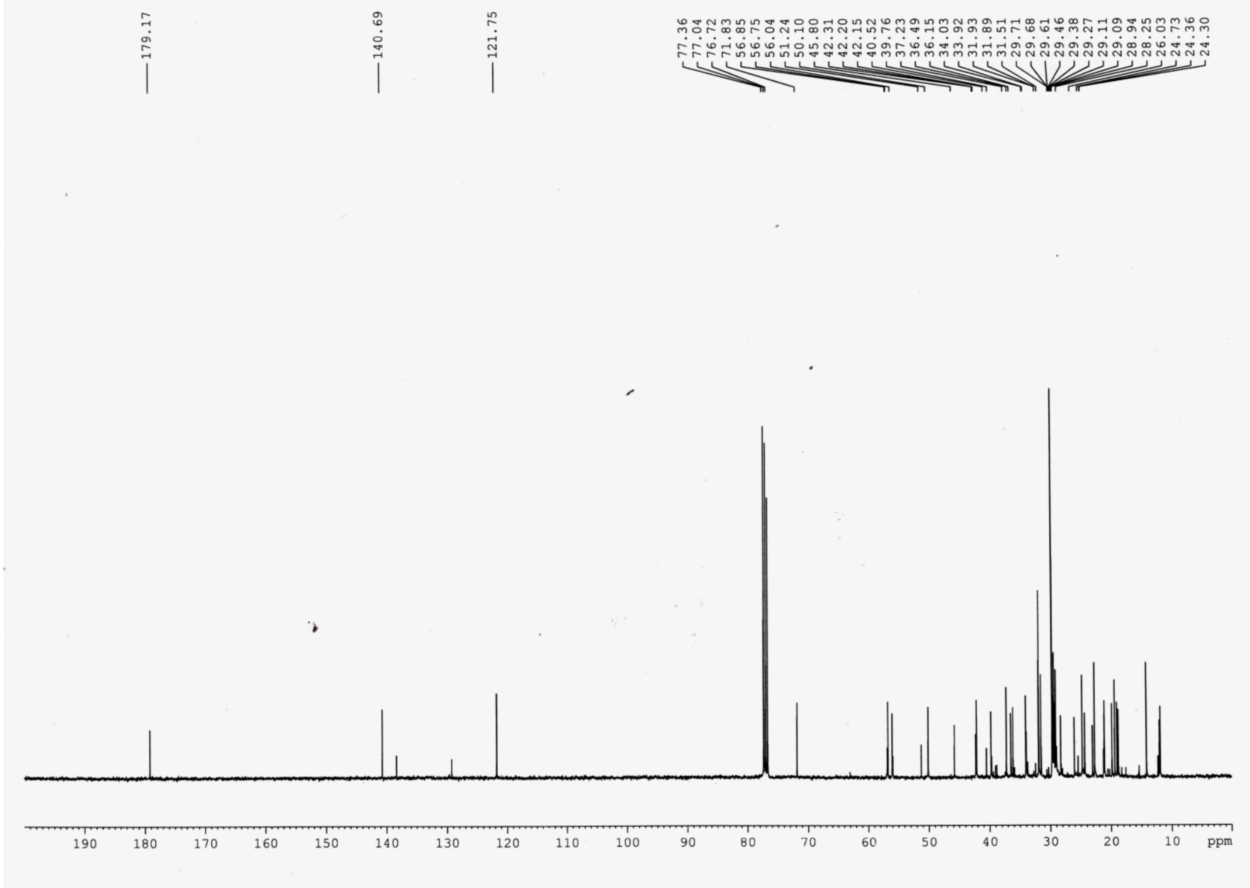


**Figure S3.** $^{13}C$ NMR spectrum of *Corchorus capsularis* L. leaf derived β-sitosterol (β-sitosterol$_{CCL}$). The spectrum was recorded in a Bruker Avance spectrometer at 100 MHz by using $CDCl_3$ as solvent system.

**Figure S4.**

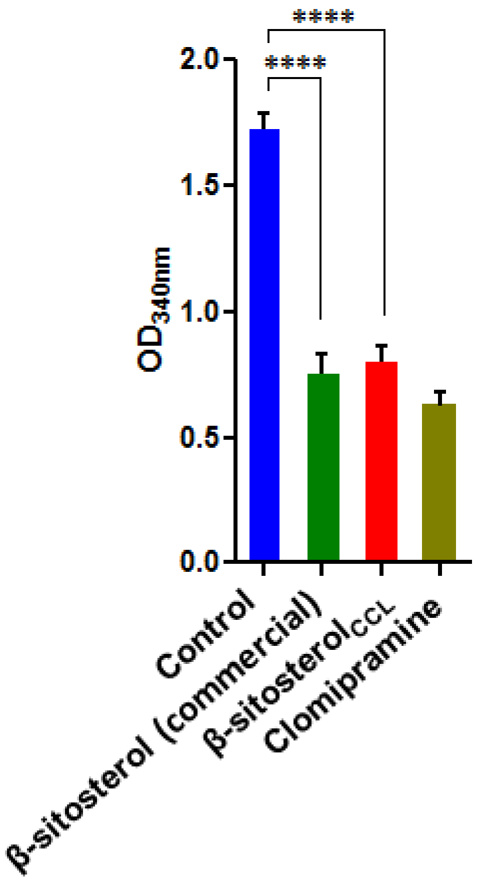


**Figure S4.** TryR assay in soluble extract of *L. donovani* promastigotes. Effect of *Corchorus capsularis* L. leaf derived β-sitosterol (β-sitosterol$_{CCL}$) on the activity of TryR enzyme in soluble extract of *L. donovani* promastigotes was evaluated by measuring NADPH consumption compared to control. Herein, commercial β-sitosterol (Abcam, USA) was used to validate the observation of β-sitosterol$_{CCL}$. Clomipramine (10 μM) was used as positive control. Results are representative of three separate experiments of mean±S.E. and statistical significance is calculated compared to control by using one-way ANOVA with Dunnett's multiple comparison test; where, ****$p < 0.0001$ is considered as statistically significant.

**Figure S5.**

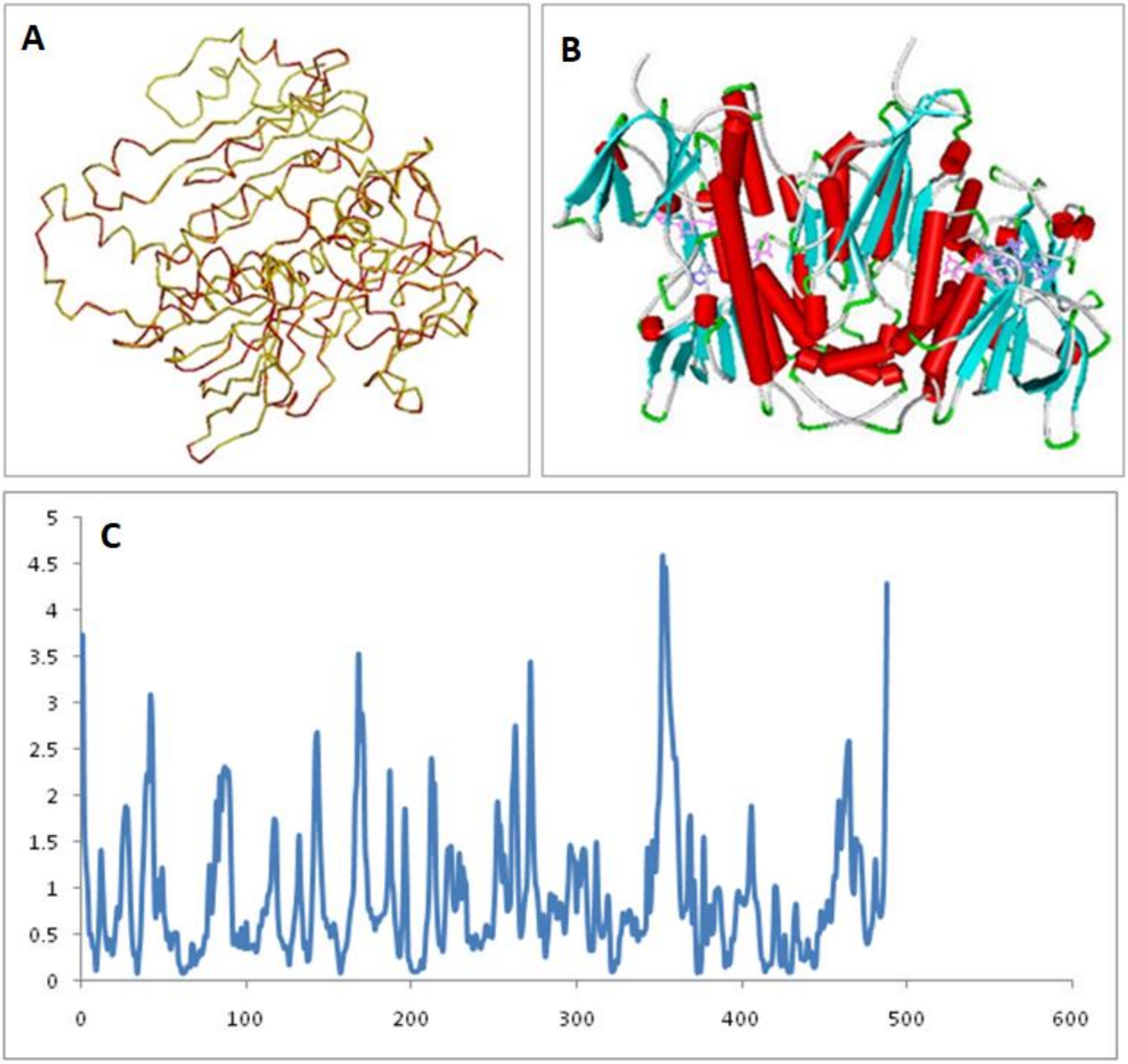


**Figure S5.** (A) Superimposition of the backbones of *Ld*TryR against the template. *Ld*TryR is presented in red and the template is presented in yellow. The RMSD of the backbone atoms of the *Ld*TryR with template was 0.25Å reflecting a good model quality. (B) Built 3D modeled structure of homodimeric *Ld*TryR along with FAD molecule (pink) and NADPH molecule (blue). (C) Extent of fluctuations of the dimeric *Ld*TryR. The most fluctuating amino acid residues are Pro42, Asp84, Asp142, Gly168, Asp272, Gly352, Ser464, Ser488.

**Figure S6.**

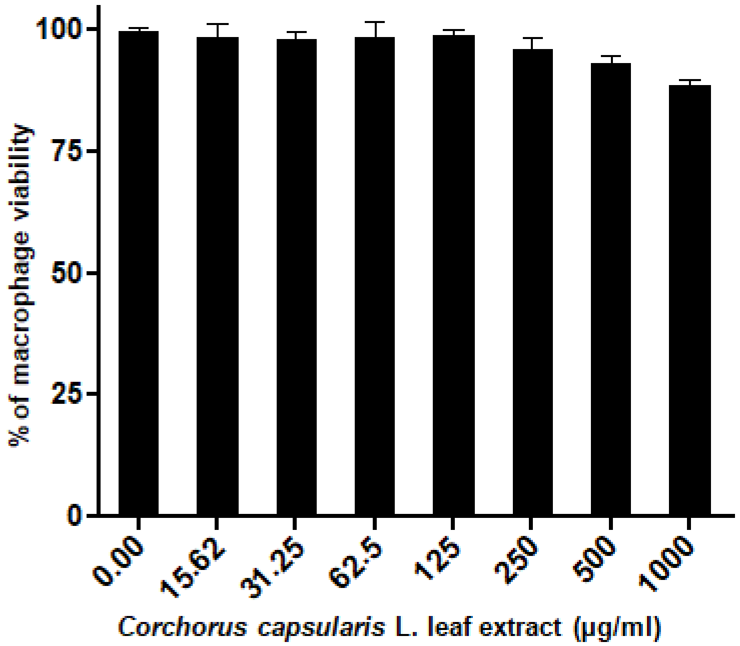


**Figure S6.** Cytotoxic effect of *Corchorus capsularis* L. leaf extract against murine RAW 264.7 macrophages. Macrophages ($1\times10^5$/ml) were treated with increasing concentration of the extract (0-1000 µg/ml) for 48 h, MTT assay was performed and 11.29±0.37% cells were found to be affected with highest dose (1000 µg/ml) reference to control. The results are expressed herein from three independent experiments as mean ± S.E.

**Figure S7.**

```
tr|Q26970|Q26970_TRYCR      -------VVIGAGSGGLEAAWNAATLYKKRVAVIDVQMVHGPPFFSALGGTCVNVGCVPK 53
tr|A4H480|A4H480_LEIBR      MPRAYDLVVLGAGSGGLEAGWNAASIYNKKVAVVEAQKEHGPPCFAALGGTCVNVGCVPK 60
sp|P39050|TYTR_LEIDO        MSRAYDLVVLGAGSGGLEAGWNAAVTHKKKVAVVDVQATHGPPALVALGGTCVNVGCVPK 60
tr|A4HSF7|A4HSF7_LEIIN      MSRAYDLVVLGAGSGGLEAGWNAAVTHKKKVAVVDVQATHGPPLFAALGGTCVNVGCVPK 60
                                   **:*********.****  ::*:***::.*  **** : **************

tr|Q26970|Q26970_TRYCR      KLMVTGAQYMEHLRESAGFGWEFDRTTLRAEWKKLIAVKDEAVLNINKSYDEMFRDTEGL 113
tr|A4H480|A4H480_LEIBR      KLMVTGAQYMDLIRESCGFGWEMDRDSIRSNWKKLITAKNKVVSDINKSYTDMFENTEGL 120
sp|P39050|TYTR_LEIDO        KLMVTGAQYMDLIRESGGFGWEMDRESLCPNWKTLIAAKNKVVNSINESYKSMFADTEGL 120
tr|A4HSF7|A4HSF7_LEIIN      KLMVTGAQYMDLIRESGGFGWEMDRESLCPNWKTLIAAKNKVVNSINESYKSMFADTEGL 120
                            **********: :*** *****:** ::  :**.**:.*::.* .**:** .** :****

tr|Q26970|Q26970_TRYCR      EFFLGWGSLESKNVVNVRESADPASAVKERLETEHILLASGSWPHMPNIPGIEHCISSNE 173
tr|A4H480|A4H480_LEIBR      SFHMGFGALQDAHTVVVRKSEDPESDVLETLEADYILIATGSWPTRLGIPGDELCITSNE 180
sp|P39050|TYTR_LEIDO        SFHMGFGALQDAHTVVVRKSEDPHSDVLETLDTEYILIATGSWPTRLGVPGDEFCITSNE 180
tr|A4HSF7|A4HSF7_LEIIN      SFHMGFGALQDAHTVVVRKSEDPHSDVLETLDTEYILIATGSWPTRLGVPGDEFCITSNE 180
                            .*.:*:*:*:. :.* **:* ** * * * *::::**:*:****   .:** * **:***

tr|Q26970|Q26970_TRYCR      AFYLPEPPRRVLTVGGGFISVEFAGIFNAYKPKDGQVTLCYRGEMILRGFDHTLREELTK 233
tr|A4H480|A4H480_LEIBR      AFYLDEAPKRALCVGGGYISVEFAGIFNAYKPPDGQVDLCYRGEVILRGFDLEVRKSLMK 240
sp|P39050|TYTR_LEIDO        AFYLEDAPKRMLCVGGGYIAVEFAGIFNGYKPCGGYVDLCYRGDLILRGFDTEVRKSLTK 240
tr|A4HSF7|A4HSF7_LEIIN      AFYLEDAPKRMLCVGGGYIAVEFAGIFNGYKPCGGYVDLCYRGDLILRGFDTEVRKSLTK 240
                            **** : *:* * ****:*:*********.*** .* * *****::******  :*:.* *

tr|Q26970|Q26970_TRYCR      QLTANGIQILTKENPAKVELNADGSKSVTFESGKKMDFDLVMMAIGRSPRTKDLQLQNAG 293
tr|A4H480|A4H480_LEIBR      QLEANGIKIRTKVNPSRITKNADGSKHVCFEDGTEADYDQVMLAVGRAPRSKALQLDKAG 300
sp|P39050|TYTR_LEIDO        QLGANGIRVRTNLNPTKITKNEDGSNHVHFNDGTEEDYDQVMLAIG-VPRSQALQLDKAG 299
tr|A4HSF7|A4HSF7_LEIIN      QLGANGIRVRTNLNPTKITKNEDGSNHVHFNDGTEEDYDQVMLAIGRVPRSQALQLDKAG 300
                            ** ****:: *: **:::  * ***: * *:.*.: *:* **:*:*  **:: ***::**

tr|Q26970|Q26970_TRYCR      VMI-KNGGVQVDEYSRTNVSNIYAIGDVTNRVMLTPVAINEAAA-LVDTVFGTTPRKTDH 351
tr|A4H480|A4H480_LEIBR      VKMGKNGAVVVDAYSKTSVDNIYAIGDVTDRLMLTPVAINEGSA-FVETLFGGKPRATDH 359
sp|P39050|TYTR_LEIDO        VRTGKNGAVQVDAYSKTSVDNIYAIGDVTNRVMLTPVAINEGACVLLETVFGGKPRATDH 359
tr|A4HSF7|A4HSF7_LEIIN      VRTGKNGAVQVDAYSKTSVDNIYAIGDVTNRVMLTPVAINEGAA-FVETVFGGKPRATDH 359
                            *   ***.* ** **:*.*.*********:*:**********.:. :::*:** .** ***

tr|Q26970|Q26970_TRYCR      TRVASAVFSIPPIGTCGLIEEVASKRYEVVAVYLSSFTPLMHKVSGSKYKTFVAKIITNH 411
tr|A4H480|A4H480_LEIBR      TKVACAVFSIPPIGTCGLTEEEAAKKYDVVAVYESSFTPLMHNISGSKHKTFMIRIVTKE 419
sp|P39050|TYTR_LEIDO        TKVACAVFSIPPIGTCGMTEEEAAKNYETVAVYASSFTPLMHNISGSKHKEFMIRIITNE 419
tr|A4HSF7|A4HSF7_LEIIN      TKVACAVFSIPPIGTCGMTEEEAAKNYETVAVYASSFTPLMHNISGSKHKEFMIRIITNE 419
                            *:**.************: ** *:*.*:.**** ********::****:* *: :*:*:.

tr|Q26970|Q26970_TRYCR      SDGTVLGVHLLGDNAPEIIQGIGICLKLNAKISDFYNTIGVH------------------ 453
tr|A4H480|A4H480_LEIBR      KDGEVLGVHMLGDSAPEIIQSVGICMRMGAKISDFHSTIGVHPTSAEELCSMRTPAYFYE 479
sp|P39050|TYTR_LEIDO        SNGEVLGVHMLGDSAPEIIQSVGICMKMGAKISDFHSTIGVHPTSAEELCSMRTPAYFYE 479
tr|A4HSF7|A4HSF7_LEIIN      SNGEVLGVHMLGDSAPEIIQSVGICMKMGAKISDFHSTIGVHPTSAEELCSMRTPAYFYE 479
                            .:* *****:***.******.:***:::.******:.*****

tr|Q26970|Q26970_TRYCR      ------------                                    453
tr|A4H480|A4H480_LEIBR      KGKRVEKLSCNL                                    491
sp|P39050|TYTR_LEIDO        SGKRVEKLSSNL                                    491
tr|A4HSF7|A4HSF7_LEIIN      SGKRVEKLSSNL                                    491
```

**Figure S7.** Multiple sequence alignment method. Multiple sequence alignment was performed by using the amino acid sequences of the proteins *LiTryR* from *Leishmania infantum* TryR, *Tc*TryR from *Trypanosoma cruzi* TryR and *Lb*TryR from *Leishmania braziliensis* TryR. The identified active site amino acid residues are marked in red.

## 4. Supplementary Tables

**Table S1.** Description and effect of fractions (F1-F13) on *L. donovani* promastigotes.

| Major Fraction | Pooled fraction | [a]$IC_{50}$ (μg/ml) |
|---|---|---|
| F1 | 1-15 | 58.4±2.14 |
| F2 | 16-65 | 47.3±1.67 |
| F3 | 66-84 | 105.6±4.91 |
| F4 | 85-89 | 17.7±0.43 |
| F5 | 90-91 | 21.43±0.82 |
| F6 | 92-109 | 37.2±1.60 |
| F7 | 110-143 | 31.2±1.70 |
| F8 | 144-171 | 55.5±2.05 |
| F9 | 172-195 | 72.8±0.78 |
| F10 | 196-208 | 141.6±1.76 |
| F11 | 209-230 | >200.0 |
| F12 | 231-308 | 91.8±2.87 |
| F13 | 309-441 | >200.0 |

[a]The concentration of fractions that inhibited 50% growth of the *L. donovani* promastigotes. Results are depicted herein as mean ± S.E. of three different independent experiments.

**Table S2.** List of amino acid residues of homodimeric *Ld*TryR with their binding interaction energy values with the ligand β-sitosterol (β-sitosterol$_{CCL}$) in presence of FAD and NADPH. The first letter represents the chain ID followed by the three letter code of the amino acid and then the residue number of the amino acid of *Ld*TryR. Marked in red are the active site amino acid residues.

| Residue | Interaction Energy (kcal/mol) |
|---|---|
| A_GLY459 | -1.053530 |
| A_VAL460 | -1.626960 |
| A_HIS461 | -3.616330 |
| A_PRO462 | -0.970229 |
| A_THR463 | -0.196896 |
| B_THR51 | -3.366660 |
| B_CYS52 | -3.030530 |
| B_VAL55 | -2.448090 |
| B_GLY56 | -2.219040 |
| B_CYS57 | -2.952030 |
| B_LYS60 | -0.599220 |
| B_SER162 | -1.493710 |
| B_TRP163 | -1.055020 |
| B_PRO164 | -2.029550 |
| B_THR177 | -0.316481 |
| B_SER178 | -2.057590 |
| B_ASN179 | -1.536950 |
| B_PHE182 | -0.675878 |
| B_TYR198 | -3.227310 |
| B_ILE199 | -2.034010 |
| B_GLU202 | -1.991880 |
| B_PHE203 | -0.300822 |
| B_MET282 | -0.137236 |
| B_ALA284 | -0.101688 |
| B_ILE285 | -0.587216 |
| B_GLY286 | -0.369577 |
| B_VAL287 | -0.150055 |
| B_ASP326 | -0.828915 |
| B_MET332 | -1.991700 |
| B_LEU333 | -3.486890 |
| B_THR334 | -2.358500 |
| B_PRO335 | -1.878500 |
| B_VAL362 | -0.047897 |
| B_ALA363 | -1.868340 |
| B_CYS364 | -1.914780 |
| B_ALA365 | -3.463620 |
| B_VAL366 | -0.437207 |
| B_PHE367 | -1.244690 |

## 5. Supplementary References

1. Pramanik, P.K., Paik, D., Pramanik, A. & Chakraborti, T. White jute (Corchorus capsularis L.) leaf extract has potent leishmanicidal activity against Leishmania donovani. *Parasitol. Int.* **71**, 41-45 (2019).

2. Das, P., Paik. D., Naskar, K. & Chakraborti, T. Leishmania donovani serine protease encapsulated in liposome elicits protective immunity in experimental visceral leishmaniasis. *Microbes Infect.* **20**, 37-47 (2018).

3. Weidenfeld, I. et al. Homogentisic acid-derived pigment as a biocompatible label for optoacoustic imaging of macrophages. *Nat. Commun.* **10**, 5056 (2019).
